# E-cigarette vaping is associated with pro-fibrotic gene expression in kidney and liver tissues

**DOI:** 10.1007/s00109-026-02699-1

**Published:** 2026-07-31

**Authors:** Wanjun Gu, Howard Chang, Poorvi Saini, Samvel Gaboyan, Jarod Olay, Jorge A. Masso-Silva, John Shin, Ira Advani, Ashley Du, Cameron Brand, Joan Heller Brown, Alexia Perryman, Laura E. Crotty Alexander

**Affiliations:** 1 Department of Neurology, Weill Institute for Neurosciences, University of California San Francisco, San Francisco, CA 94158 USA; 2 Pulmonary and Critical Care Section, Medicine Section, VA San Diego Healthcare System, La Jolla, CA 92161 USA; 3 Division of Pulmonary, Critical Care and Sleep Medicine and Physiology, Department of Medicine, University of California, San Diego, La Jolla, CA 92093 USA; 4 Department of Pharmacology, University of California, San Diego, La Jolla, CA 92093 USA

**Keywords:** Fibrosis, E-cigarette, Tobacco, Kidney, Liver, Transcriptomics

## Abstract

Conventional tobacco use causes a wealth of diseases and adversely affects cells and organ systems across the body. The long-term effects of e-cigarette vaping on the same remain unclear. Identifying early pathogenic signals at the organ level via animal models may shed light on potential downstream effects in humans. Here we investigate transcriptomic changes in the kidney and liver, organs known to be damaged by long-term combustible tobacco use, of mice exposed daily to e-cigarette aerosols (vapor) with or without nicotine. C57BL/6 male 6–8 week-old mice underwent whole-body exposure to room air, e-cigarette (3rd generation box mod) vapor containing 70:30 propylene glycol and glycerin (PG: Gly) without nicotine (Vehicle), and e-cigarette vapor with 6 mg/mL freebase nicotine in 70:30 PG: Gly (E-cig) for 1 h daily for 3 months. RNA sequencing on kidney and liver tissues and apriori gene set analysis were performed. Unbiased principal component analysis identified closer clustering of E-cig and Vehicle groups, relative to Air, in the kidney. Assessment of the a priori gene set found nicotine to be associated with greater transcriptomic changes in the kidney while vehicle chemicals induced greater changes in the liver. Alterations in expression of *Car3* and *Foxo3* identify oxidative stress and inflammation in the renal system associated with e-cigarette exposure, while dysregulated *Il6ra* and *Lpin1* in the liver highlight disruptions in lipid metabolism and immune signaling. Chronic inhalation of e-cigarette vapor alters gene expression in downstream organs, in a pattern most consistent with promotion of fibrosis and metabolic dysregulation, underscoring the need to define long-term pathophysiologic effects of e-cigarette vaping across the body.

**Supplementary Information:**

The online version contains supplementary material available at 10.1007/s00109-026-02699-1.

## Key Message

- Chronic inhalation of e-cigarette vapor alters gene expression in kidneys and liver.
- Oxidative stress and inflammatory gene expression was altered in the kidneys.
- E-cigarettes led to disruptions in expression of lipid metabolism and immune signaling genes in the liver.
- E-cigarette exposure promotes genes associated with fibrosis and metabolic dysregulation.

**Supplementary Information:**

The online version contains supplementary material available at 10.1007/s00109-026-02699-1.

## Introduction

**Fig. 1:**
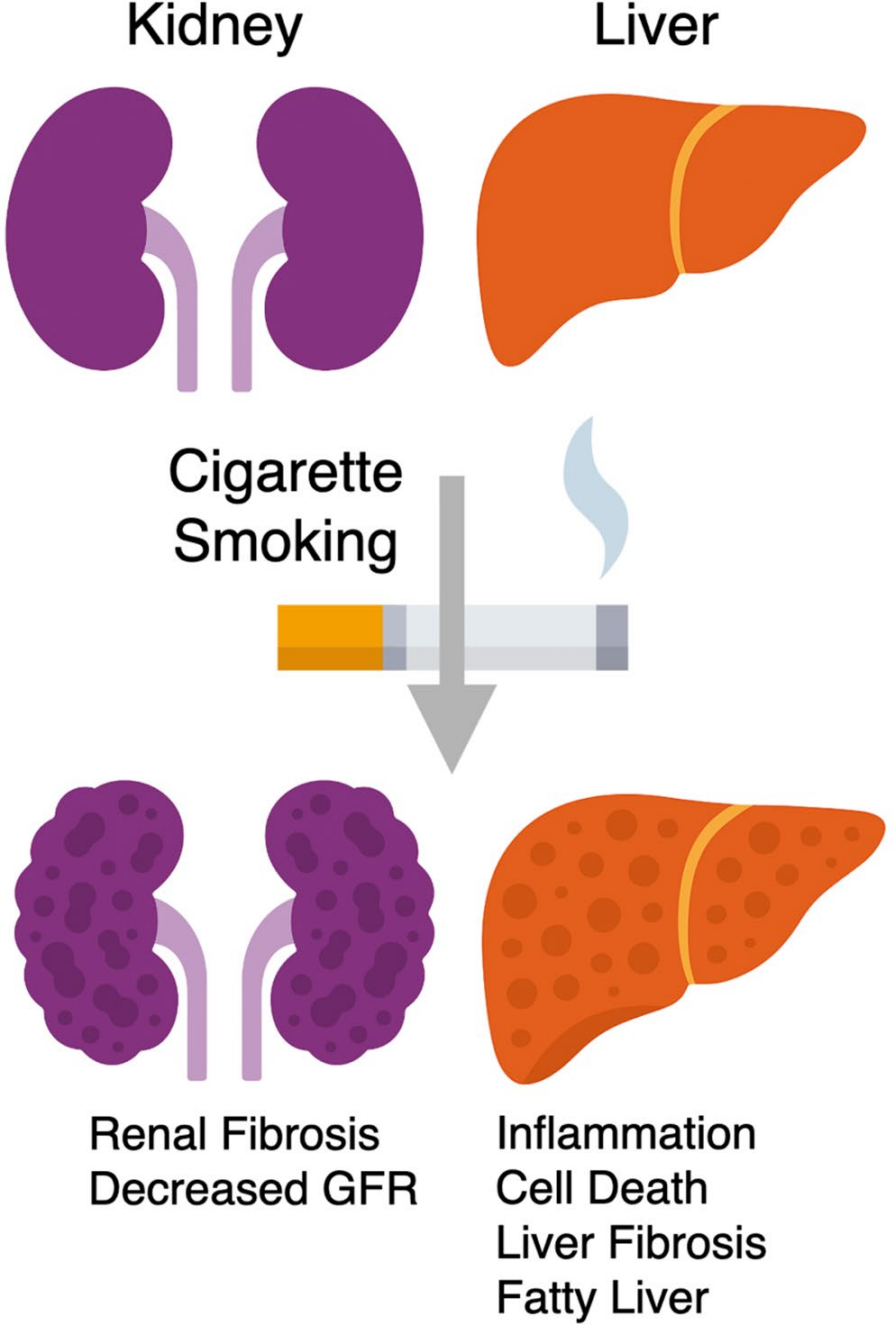
Chronic inhalation of tobacco smoke is known to adversely affect both the renal and hepatic organ systems, leading to organ fibrosis and diminished function

Inhalation of combustible tobacco smoke is well established as a cause of numerous adverse health effects, including hepatorenal toxicity [1–4]. Specifically, exposure to combustible tobacco smoke is associated with elevated rates of renal failure [1, 5, 6], renal fibrosis [3, 7, 8], liver failure [9] and liver fibrosis [10, 11]. These organ-damaging effects are thought to arise via tissue injury caused by the 4,000–7,000 toxic chemicals present in tobacco smoke, subsequently complicated by fibrosis during tissue repair [4] (Fig. 1). Although the molecular, cellular and tissue-level effects of combustible tobacco use are well characterized, electronic (e)-cigarettes and vaping devices represent a newer form of nicotine delivery for which the full spectrum of health effects remains to be determined.

Because e-cigarettes have only been in regular use since the mid 2010s, it will be many more years before we identify health effects of chronic vaping via epidemiologic methods. Murine models have yielded helpful data regarding the impact of daily e-cigarette aerosol (commonly called vapor) inhalation, including findings that e-cigarette vaping alters responses to respiratory pathogens [12–14], alters cardiopulmonary physiology [15–17], and leads to gene expression changes across the body [18–24]. Few studies have focused on the potentially renal toxic effects of e-cigarettes. However, the data thus far indicates that e-cigarette vaping does cause kidney damage and diminishes renal function [25]. In our previous studies, we found that “vape pen” (2nd generation) e-cigarette aerosol exposures 1 h daily for 3–6 months increased circulating proinflammatory and pro-fibrotic proteins, decreased renal filtration rates, and induced fibrosis in the kidneys and livers of female mice, both in C57BL/6 and CD-1 backgrounds [24]. Another study found that chronic exposure to nicotine containing e-cigarette vapor in the setting of a high-fat diet increased inflammatory responses, oxidative stress-induced DNA injury, and pro-fibrotic markers in mouse kidneys, suggesting accelerated development of renal pathology [26]. Interestingly, no-nicotine (Vehicle) also led to renal pathology, but via suppression of mitochondrial OXPHOS complexes and extracellular matrix deposition, which is likely to cause structural instability.

Regarding hepatic effects of e-cigarettes, one study utilizing a murine model found that nicotine containing e-cigarette exposure was associated with hepatic steatosis in adult offspring, whereas non-nicotine e-cigarette exposure (Vehicle) led to metabolic alterations and liver damage in both dams and offspring [27]. An in vitro study of Kupffer cells exposed to e-cigarette extracts identified robust inflammatory responses, oxidative stress and cytokine release, though the physiologic relevance of these findings is inherently limited by the cell culture system [28]. Both studies employed a similar generation of e-cigarette (3rd ) and nicotine type (base nicotine) and concentration (6–18 mg/mL) as the current study. A separate study of e-cigarette flavorant chemicals demonstrated that vanillin, ethyl vanillin, and ethyl maltol induced cytotoxicity in HepG2 cells, raising concern for possible hepatotoxicity in human vapers [29] . Collectivly, these findings suggest that multiple e-cigarette constituents promote hepatic inflammation, a recognized early step in the pathogenesis of liver fibrosis, or direct cellular injury, both of which can initiate or amplify fibrogenic cascades. Notably, nicotine containing e-cigarette exposures appear to produce distinct pathological profiles compared to Vehicle (no nicotine) exposures, underscoring the specific contribution of nicotine to vaping-associated hepatotoxicity.

The vast majority of e-cigarettes and e-liquids currently on the market have not been thoroughly evaluated for health effects, particularly for adverse outcomes beyond the lung. To characterize the impact of long-term, daily inhalation of e-cigarette aerosols on renal and hepatic gene expression, as a molecular precursor to the structural and functional pathology we have previously identified, mice were exposed daily for 12 weeks to e-cigarette aerosols generated from chemicals common in all e-liquids: propylene glycol, glycerin, and nicotine. To further delineate the respective contributions of nicotine versus other e-cigarette constituents, animals were assigned to one of two exposure groups: e-cigarette aerosol containing nicotine (E-cig) or nicotine-free aerosol (Vehicle).

## Methods

### Murine E-cigarette exposures

C57BL/6 6–8 week-old male mice (Harlan) were exposed to e-cigarette aerosols or air for 60 min daily, 5 days/week, for 12 weeks using the whole-body SCIREQ InExpose System (Emka). Whole-body exposure was chosen over nose-only to minimize stress-induced changes in immunity and inflammation. Prior to starting exposures, mice were acclimated to the whole-body exposure chambers for 30 min daily for 2 days. Nicotine (freebase) containing e-liquid consisted of 70% propylene glycol (PG), 30% glycerin (Gly), and 6 mg/ml base nicotine (Sigma), to mimic constituents of 3rd generation e-devices. Vehicle mice were exposed with 70:30 PG: Gly e-liquid without nicotine, while control mice were exposed to environmental air alone. Aerosols were generated with an e-cigarette Box Mod device with a standard tank (1.8 Ω) and AC power. Pneumatic pressure and puff topography was modeled on human e-cigarette vapers and mirrored our previous studies [18, 24]. In brief, e-cigarettes were activated 3 times per minute for 4 s at 2 L/min, followed by room air for 16 s. Mice were placed in prewarmed cages for 30 min after exposures. After the final exposure, mice were euthanized and kidney and liver tissues were harvested with half placed into 4% paraformaldehyde at 4 °C and half snap frozen and stored at -80 °C. Fixed tissues were transferred into PBS at 24 h, paraffin embedded, sliced and stained with H&E and Masson’s Trichrome. Frozen tissue underwent total RNA isolation (Qiagen) followed by bulk RNAseq (Illumina NovaSeq X Plus). All animal experiments were conducted in accordance with the National Institutes of Health *Guide for the Care and Use of Laboratory Animals* under protocols approved by the Institutional Animal Care and Use Committee at the University of California San Diego.

### Quantification of structural and fibrotic changes

Quantification analysis of collagen in Masson Trichrome stained tissue was done using QuPath software. First, the calculate intensity feature was used to quantify the intensity of blue stain present in each organ tissue section. The calculate intensity tool identified and outlined/annotated all detectable cells in each microscopy image and calculated the intensity of blue stain present yielding mean gray value (ratio of pixel area of the stain of interest to the total pixel area of the image). Mean gray values to assess for collagen content differences in kidneys and livers between Air (control), Vehicle and E-cig (vehicle + nicotine) exposures were analyzed via non-parametric Kruskal-Wallis test (GraphPad Prism).

### RNA extraction

Liver and kidney samples were weighed, sectioned into 2–4 mm^3^ pieces, 600 µl Trizol added and tissue homogenizer applied. The homogenizer was cleaned with RNAse away, 70% ethanol, RNAse away, and RNAse/DNAse free water in-between samples. Ethanol (600 uL) was added and samples centrifuged at 15,000 rpm for 1 min. Samples were transferred to Zymo Spin columns, 80 uL of DNAse I Reaction Mix (Qiagen) added and samples incubated at room temperature for 15–20 min. RNA Prep Buffer (400uL) was added and samples centrifuged for 1 min. RNA Wash Buffer (700uL) was added to each column and samples were centrifuged for 1 min. After another RNA Wash Buffer (400uL) application, samples were centrifuged for 2 min. Column filters were transferred to new RNAse free tubes and centrifuged dry for 5 min. DNAse/RNAse free water (50uL) was added to each column filter, incubated at room temperature for 2 min, and centrifuged for 3 minutes. The flowthrough was re-eluted 1 time and the concentration of RNA was measured (Nanodrop), prior to submission of samples to the UC San Diego IGM Core for transcriptomics (1.25B reads per lane, Illumina NovaSeq X Plus).

### RNA sequencing analysis

RNA sequencing libraries were prepared using 50-base-pair single-end reads with an average sequencing depth of approximately 50 million reads per sample. The raw sequencing reads were aligned to the mouse reference genome (GRCm39, Ensembl release 112) using the Bowtie version 1.3.0 aligner and quantified with RSEM version 1.3.0 [30]. Gene annotations were derived from a comprehensive mouse gene annotation dataset provided by the GENCODE project [31]. This dataset includes detailed information about known protein-coding genes, non-coding genes, and their transcript variants in the mouse genome, enabling accurate mapping of sequencing reads to specific genomic features. To reduce noise in the dataset, only genes with counts per million (CPM) values greater than 100 in at least two samples were retained. This filtering ensured that only genes with sufficient expression levels across samples were included in the analysis.

Normalization of gene expression data was performed using the Trimmed Mean of M-values (TMM) method as implemented in the edgeR R package, which calculates scaling factors for each library by trimming the most extreme log-fold changes and log-abundance values, then computing a weighted mean of the remaining ratios. This approach accounts for differences in both library size and RNA composition across samples, effectively adjusting for sample-to-sample variability while preserving biological differences.

Differential gene expression (DGE) analysis was conducted separately for kidney and liver tissues, each using an independent data object containing only the samples from that tissue (kidney: *n* = 17; liver: *n* = 17). Exact tests were used for pairwise comparisons between experimental groups within each tissue. Genes were considered significantly differentially expressed if their adjusted p-values, corrected using the false discovery rate (FDR), were less than or equal to 0.05, and their absolute log-fold change was greater than or equal to 1.

### A priori gene set analysis

To enhance statistical power, a separate analysis was conducted on a curated a priori gene set specific to liver and kidney fibrosis, sourced from published literature and created prior to the generation of transcriptomic data (Supplementary Tables 1–3). The kidney gene set contained 109 genes, and the liver gene set contained 1 gene (*Vim*). Of the 109 kidney a priori genes, 16 were detected above the expression threshold (CPM > 100 in ≥ 2 samples) and retained for analysis. For these detected genes, false discovery rate correction was applied exclusively within the a priori gene set rather than across the full transcriptome, reducing the multiple-testing burden and increasing sensitivity to true associations within the curated gene set. Genes with within-set FDR ≤ 0.05 and |log₂ fold change| ≥ 1 were considered significantly differentially expressed within the a priori context.

### Biological pathway analysis

Following differential gene expression analysis, pathway enrichment analysis was performed to contextualize systematic alterations within established biological processes. All genes with nominal significance (*p* < 0.05) in each comparison (E-cig vs. Air and VEH vs. Air for both kidney and liver tissues) were included as input. Analyses were conducted using the pathfindR R package [32], which systematically identifies active subnetworks and enriched pathways based on the Kyoto Encyclopedia of Genes and Genomes (KEGG) database [33]. Pathways with adjusted p-values < 0.05 were considered significantly enriched. To reduce redundancy and improve interpretability, functionally similar pathways were clustered based on gene overlap, and representative pathways from each cluster were retained for reporting.

### Data visualization

All analyses and visualizations were conducted using R v4.2.1. Principal component analysis (PCA) was performed on normalized gene expression data to evaluate clustering patterns across experimental groups. Volcano plots were generated to display -log10 transformed p-values against LogFC values of the a priori gene set, with significant genes highlighted. Scatter plots were made to depict associations between fold changes of gene expression in liver and kidney tissues.

## Results

### Kidney and liver histology

**Fig. 2:**
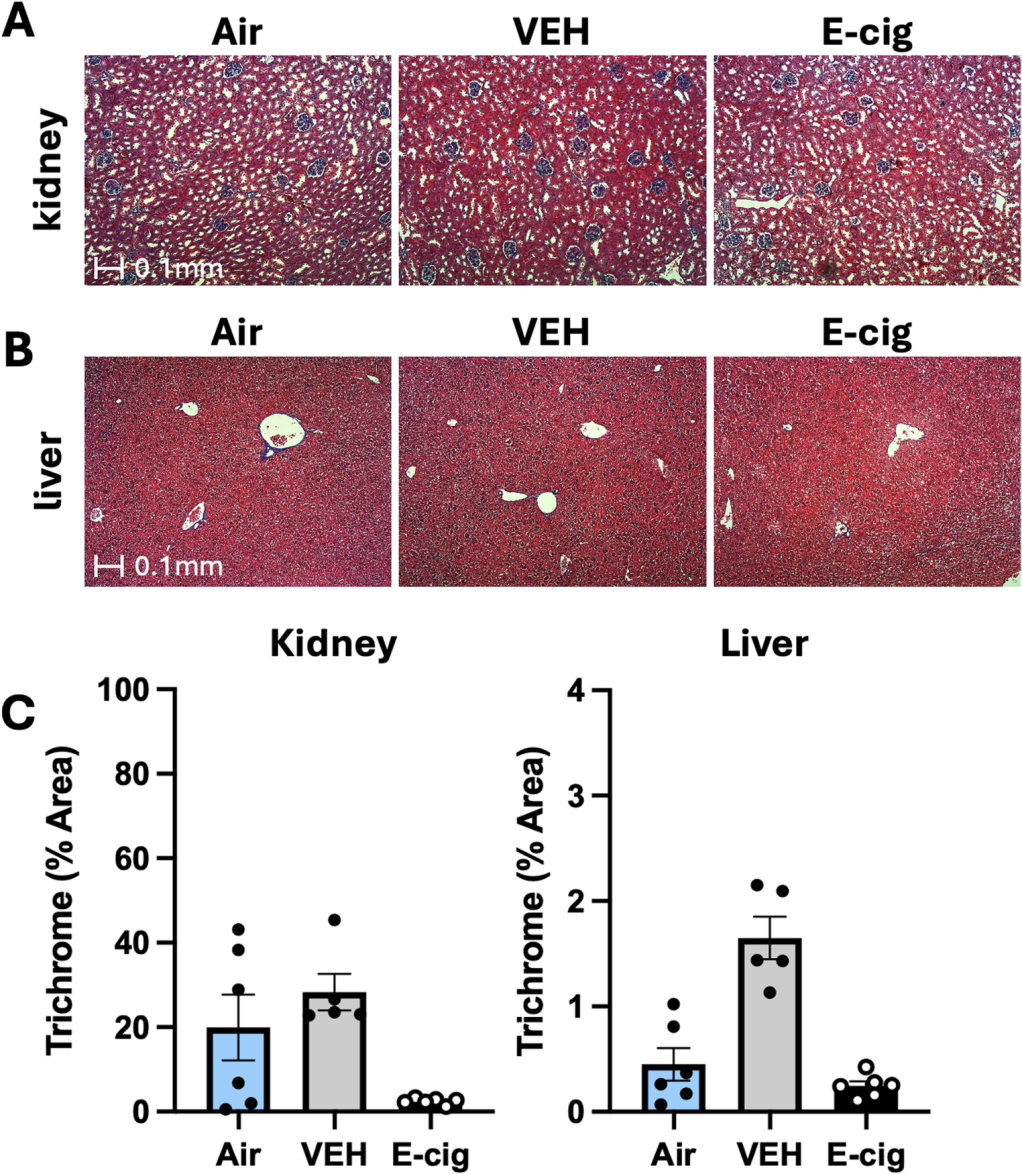
Blinded histologic assessment of trichrome stained kidney and liver tissue found no difference in collagen levels between exposed and unexposed mice. Representative images of trichrome stained kidney (**A**) and liver (**B**) tissue from controls (Air), no nicotine (VEH) aerosol exposed mice, and nicotine-containing e-cigarette (E-cig) aerosol exposed. No differences were found in collagen content by blinded histologic review (**C**). *n* = 5–6

There were no structural differences in liver and kidney tissue from Air, VEH and E-cig exposed mice (Fig. 2A-B). There were also no differences in levels of collagen, as a marker of tissue fibrosis as assessed by trichrome staining, within liver and kidney tissue from Air, VEH and E-cig exposed mice (Fig. 2C).

### Kidney and liver transcriptomic profiles

**Fig. 3:**
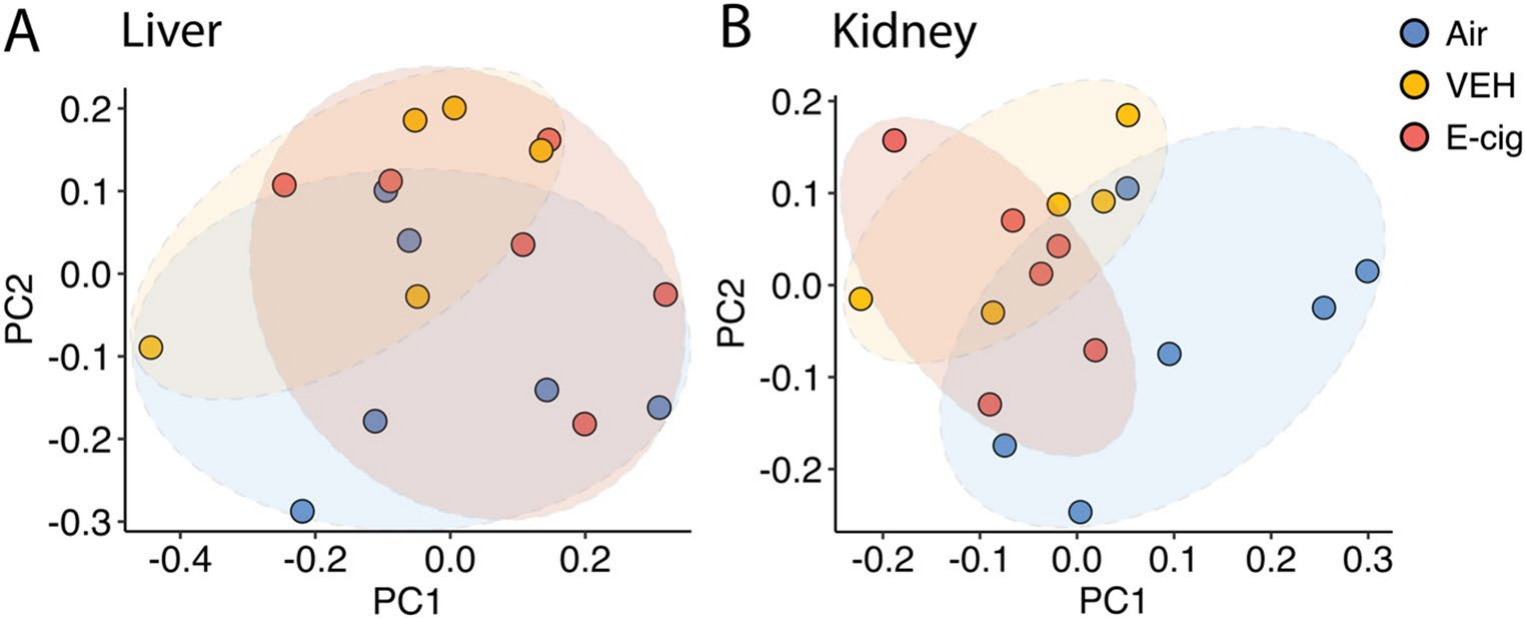
Principal Component Analysis (PCA) of gene expression profiles in liver and kidney tissues in the setting of chronic exposure to e-cigarette vapor. **(A)** PCA plot of gene expression data from liver tissue. Each point represents a sample, and the clustering reflects variation in the overall transcriptomic profile across the three experimental conditions: Air-exposed (blue), vehicle control (VEH, yellow), and e-cigarette vapor-exposed (E-cig, red). Principal Component 1 (PC1) and Principal Component 2 (PC2) account for most of the variance in gene expression among samples. Clustering patterns highlight the similar transcriptomic profiles across groups. **(B)** PCA plot of gene expression data from kidney tissues. The separation along PC1 and PC2 indicates condition-specific differences in transcriptomic profiles, with the E-cig and VEH groups having some separation in clustering away from the Air group. *n* = 5–6 per group

Principal component analysis (PCA) plots highlight global transcriptomic patterns across exposure conditions in liver and kidney tissues. Global gene expression in liver tissue from E-cig exposed mice was similar to that from Air and VEH (no nicotine) groups, indicating a lack of broad transcriptomic changes driven by nicotine or other e-cigarette vapor chemicals (Fig. 3A). In the kidney, tissue gene transcription in E-cig and VEH exposed mice predominantly clustered separately from Air, indicating transcriptomic changes driven by inhaled chemicals with e-cigarette vapor (Fig. 3B). The overlap between E-cig and VEH gene expression patterns demonstrates that non-nicotine chemicals may be the primary drivers in kidney tissue (Fig. 3B).

**Fig. 4:**
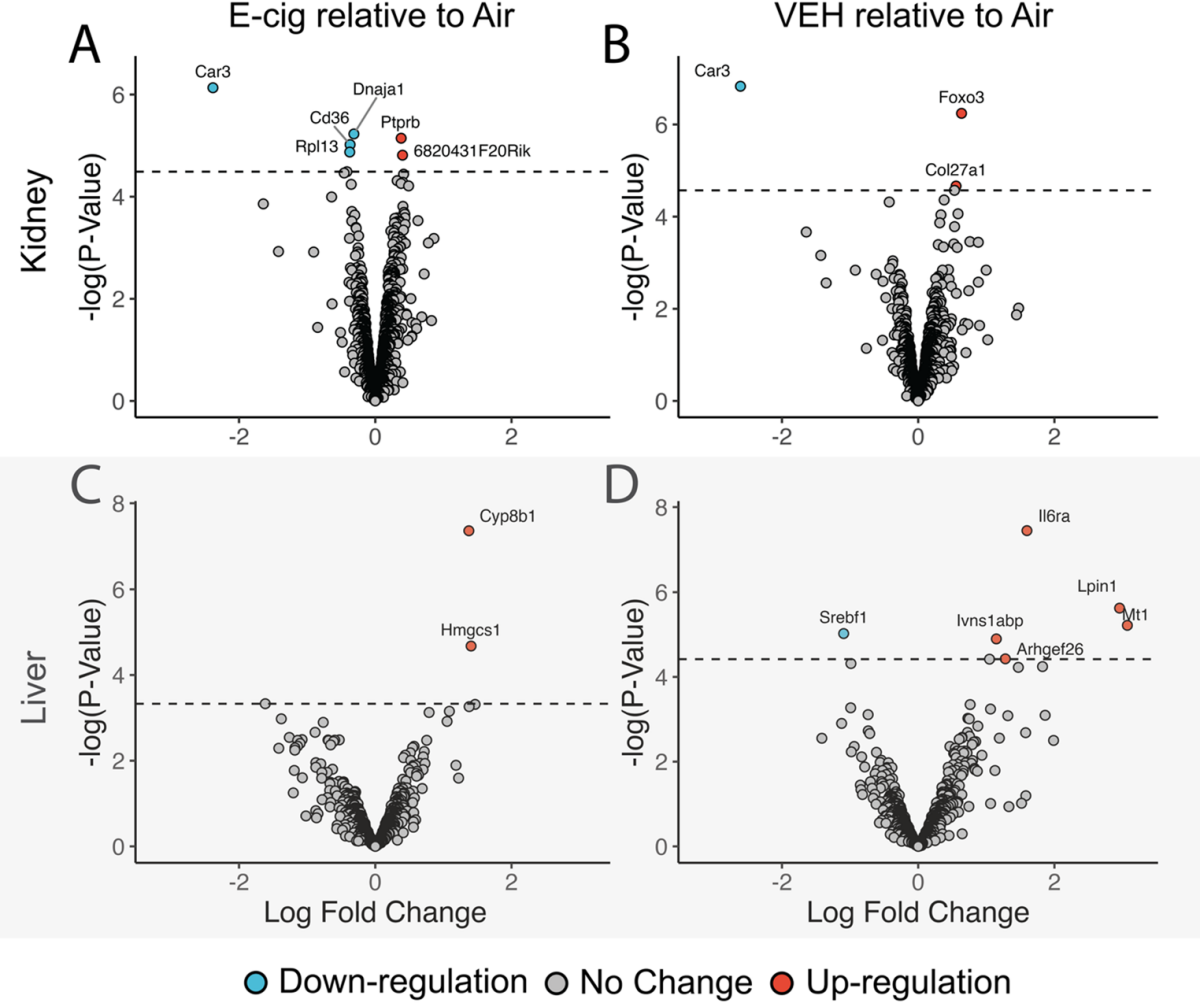
Differential gene expression in liver and kidneys of mice exposed chronically to e-cigarette vapor. Volcano plots showing the results of differential gene expression (DGE) analyses in kidney (upper panels **A **and** B**) and liver (bottom panels **C** and** D**) tissues when comparing e-cigarette vapor (E-cig, left panels **A **and** C**) and vehicle (VEH, right panels **B **and** D**) exposures, all analyzed relative to tissues from air control mice. Each point represents an individual gene, with the x-axis indicating the log fold change (LogFC) and the y-axis showing the -log10 transformed p-value. Genes with adjusted p-values (FDR) ≤ 0.05 and absolute LogFC ≥ 1 are considered significantly differentially expressed. The dashed horizontal line represents the significance threshold at -log10(p-value) corresponding to FDR = 0.05. This significance threshold was established using a false discovery rate (FDR). The FDR differs for each comparison, based on the distribution of the statistics, such that it can be ensured that genes above the FDR threshold have p-values that are truly significant after the FDR for their own comparison is used for the correction. Genes are classified as significantly down-regulated (blue), significantly up-regulated (red), or not significantly differentially expressed (grey). These plots highlight distinct transcriptomic changes induced by E-cig and VEH exposures, with differences observed between tissues (**A&B** versus **C&D**) and exposure types (**A&C** versus **B&D**). Upregulated and downregulated genes are more prominent under E-cig exposure (**A&C**; containing nicotine), indicating a stronger transcriptomic response compared to VEH (**B&D**; no nicotine)

Chronic E-cig exposure was associated with the differential expression of 10 genes in kidney tissue (FDR ≤ 0.05; Fig. 4A), whereas VEH exposure was associated with differential expression of three genes (Fig. 4B), out of 1,978 genes tested in kidney tissue. One gene was downregulated in both the E-cig and VEH conditions relative to Air, *Car3*, indicating that non-nicotine components of e-cigarette vapor contribute to this transcriptional change. Other gene expression changes included upregulation of genes associated with extracellular matrix organization, renal fibrosis, and immune regulation, consistent with molecular pathways involved in kidney fibrosis and inflammation, though these represent exploratory transcriptomic associations that warrant experimental validation (Table 1).

**Table 1:** Summary of significantly differently, regulated genes by organ and treatment group. Changes associated with e-cigarette vapor containing nicotine (EV) are indicated in bold blue, while those associated with vehicle (VEH) are in black font

Organ	Treatment	Gene Name	Symbol	LogFC	*P*-Value	FDR
Kidney	E-cig (with nicotine)	ENSMUSG00000027559	Car3	-2.38	7.37E-07	1.46E-03
		ENSMUSG00000102307	Gm38194	0.51	8.86E-07	1.75E-03
		ENSMUSG00000113673	Gm47486	0.55	1.18E-06	2.33E-03
		ENSMUSG00000028410	Dnaja1	-0.31	5.91E-06	1.17E-02
		ENSMUSG00000020154	Ptprb	0.38	7.17E-06	1.41E-02
		ENSMUSG00000111481	Gm47652	0.48	9.33E-06	1.84E-02
		ENSMUSG00000002944	Cd36	-0.37	9.56E-06	1.89E-02
		ENSMUSG00000000740	Rpl13	-0.37	1.34E-05	2.64E-02
		ENSMUSG00000071796	6820431F20Rik	0.4	1.54E-05	3.03E-02
		ENSMUSG00000110779	Gm48271	0.47	1.85E-05	3.64E-02
	Vehicle (VEH)	ENSMUSG00000027559	Car3	-2.61	1.48E-07	2.92E-04
		ENSMUSG00000048756	Foxo3	0.64	5.72E-07	1.13E-03
		ENSMUSG00000045672	Col27a1	0.56	2.16E-05	4.28E-02
Liver	E-cig (with nicotine)	ENSMUSG00000050445	Cyp8b1	1.37	4.30E-08	5.71E-05
		ENSMUSG00000093930	Hmgcs1	1.41	2.11E-05	2.80E-02
	Vehicle (VEH)	ENSMUSG00000027947	Il6ra	1.6	3.52E-08	4.67E-05
		ENSMUSG00000105703	Gm43305	2.47	2.06E-07	2.74E-04
		ENSMUSG00000020593	Lpin1	2.96	2.38E-06	3.15E-03
		ENSMUSG00000031765	Mt1	3.07	6.05E-06	8.02E-03
		ENSMUSG00000020538	Srebf1	-1.09	9.45E-06	1.25E-02
		ENSMUSG00000023150	Ivns1abp	1.15	1.27E-05	1.67E-02
		ENSMUSG00000036885	Arhgef26	1.28	3.74E-05	4.94E-02

In the liver, chronic E-cig exposure was associated with upregulation of two genes, *Cyp8b1* and *Hmgcs1* (Fig. 4C), while VEH exposure was associated with differential expression of seven genes (Fig. 4D), out of 1,328 genes tested in liver tissue. Many of these genes are involved in metabolic regulation, lipid homeostasis, and oxidative stress pathways, consistent with a tissue-specific transcriptional response to chronic e-cigarette exposure, though these associations are exploratory and require further mechanistic investigation (Table 1).

Prior to obtaining transcriptomic data, a total of 335 genes were pre-curated in the a priori gene list, including subsets associated with liver fibrosis, kidney fibrosis, and shared biological processes between liver and kidney tissues (Supplementary Tables 1–3). Of the 109 kidney fibrosis genes in the a priori list, 16 were detected above the expression threshold and included in the within-set statistical test; none reached significance after within-set FDR correction in any kidney comparison. The liver a priori gene (*Vim*) was not detected above the expression threshold. The absence of significant findings in the formal a priori test is informative: it indicates that the studied animals had not yet developed an overtly fibrotic transcriptional state at the classical pro-fibrotic signaling gene level at the time of tissue harvest, consistent with this being an early-stage, preventive-exposure model.

Notably, *Col4a1* showed nominal significance of overexpression (*p* = 0.040; LogFC = 0.177) after E-cig exposure in the kidney, suggesting potential involvement in fibrotic processes. Given its established role as a structural component of basement membranes, this modest increase may reflect early extracellular matrix remodeling and potential involvement in fibrotic processes [34]. Per-animal normalized CPM values for all 21 significantly differentially expressed genes (Table 1) across all 34 individual mice are provided in Supplementary Tables 5, and per-animal CPM values for canonical pro-fibrotic marker genes are provided in Supplementary Table 6.

### Pathway enrichment analysis

**Fig. 5:**
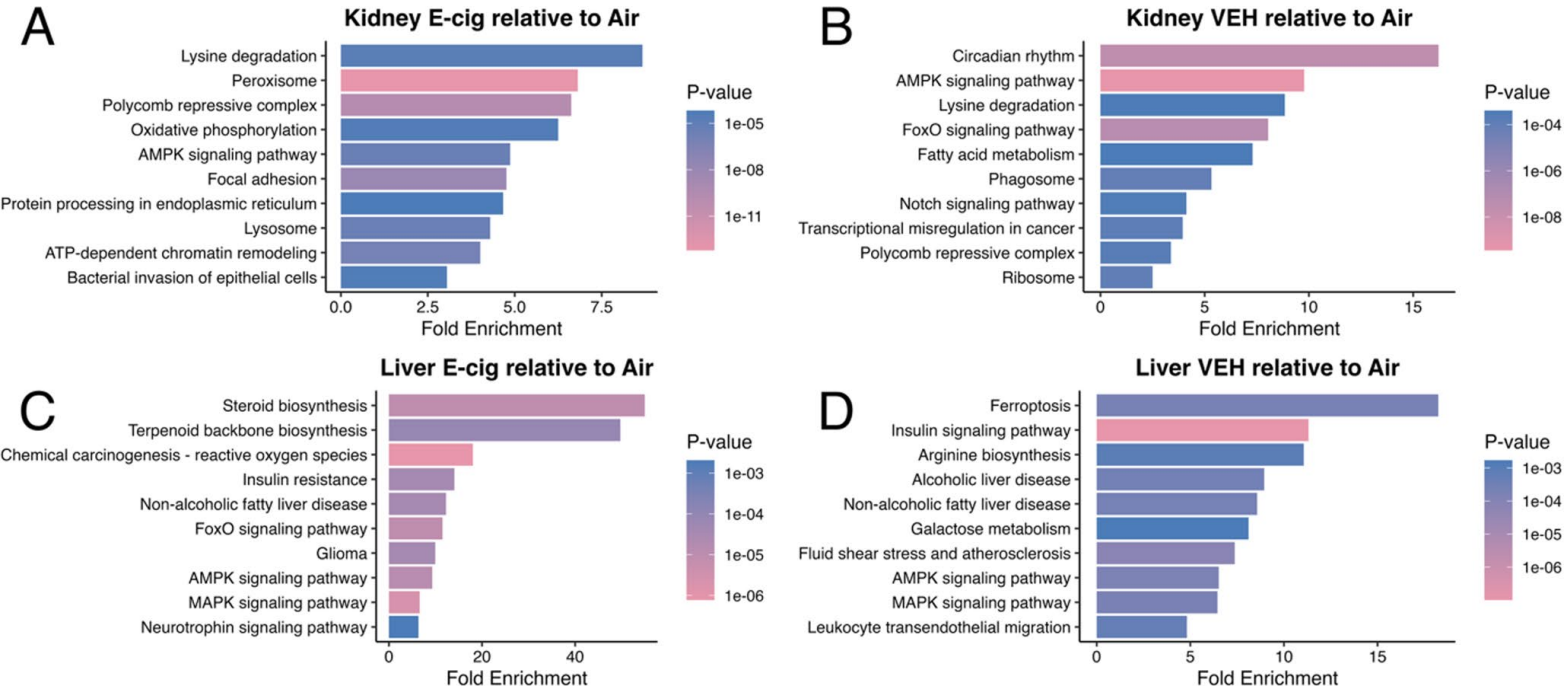
Pathway enrichment analysis of kidney and liver transcriptomes changes following chronic e-cigarette exposure. Top 10 KEGG pathway enrichment results based on fold enrichment are shown for (**A**) kidney E-cig vs. Air, (**B**) kidney VEH vs. Air, (**C**) liver E-cig vs. Air, and (**D**) liver VEH vs. Air. Bar length indicates fold enrichment, and color denotes p-value significance. E-cig exposures in the kidney were associated with enrichment of pathways related to oxidative stress, mitochondrial function, and extracellular matrix remodeling, while VEH exposures primarily affected circadian, metabolic, and signaling pathways. In the liver, E-cig exposures enriched lipid biosynthesis, insulin resistance, and ROS-related pathways, whereas VEH exposures enriched ferroptosis, insulin signaling, and fatty liver disease pathways

Pathway analysis further contextualized the transcriptomic changes induced by chronic e-cigarette vapor exposure. In the kidney, E-cig exposure enriched pathways including lysine degradation, peroxisome, oxidative phosphorylation, AMPK signaling, focal adhesion, and protein processing in the endoplasmic reticulum (Fig. 5A). In contrast, VEH exposure significantly enriched pathways related to circadian rhythm, AMPK signaling, lysine degradation, FoxO signaling, and fatty acid metabolism, indicating disrupted energy homeostasis and transcriptional control of lipid processing (Fig. 5B). These signatures point to broad perturbations in mitochondrial function, oxidative stress, and extracellular matrix interactions, suggesting that nicotine-containing aerosols amplify both metabolic and structural remodeling processes in renal tissue. In the liver, E-cig exposure produced enrichment in steroid and terpenoid backbone biosynthesis, chemical carcinogenesis (reactive oxygen species), insulin resistance, and non-alcoholic fatty liver disease (NAFLD), alongside signaling pathways including FoxO, AMPK, and MAPK (Fig. 5C). These findings suggest that nicotine-containing aerosols exacerbate metabolic dysfunction and oxidative stress, particularly through mitochondrial and ROS-driven pathways, while also disturbing cholesterol and lipid biosynthetic processes. In contrast, VEH exposure enriched pathways such as ferroptosis, insulin signaling, arginine biosynthesis, alcoholic liver disease, and NAFLD (Fig. 5D). These results highlight altered iron-dependent oxidative stress, impaired glucose and lipid metabolism, and susceptibility to hepatic injury even in the absence of nicotine. Together, these results demonstrate that both nicotine and non-nicotine e-cigarette vapor constituents disrupt central metabolic, oxidative, and stress-response pathways in kidney and liver tissues, with partially overlapping but distinct mechanistic signatures.

These pathway-level findings, each based on coordinated enrichment of ten or more genes simultaneously, provide gene-set-level evidence for the transcriptional associations described above, independent of any individual gene finding. All enriched biological pathways with fold enrichment values, adjusted p-values, and constituent up- and down-regulated genes can be found in Supplementary Table 4.

### Divergent gene expression between liver and kidney tissues

**Fig. 6:**
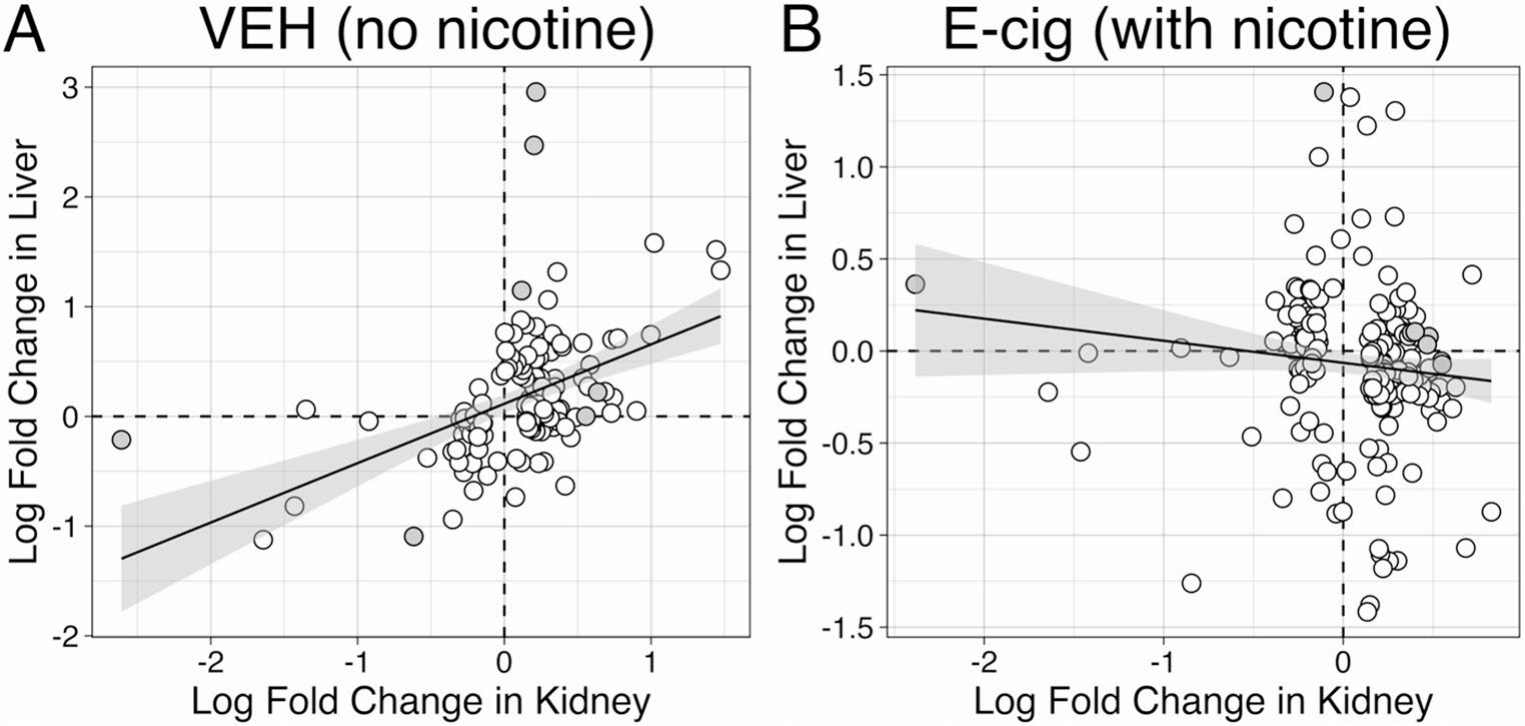
Correlation of DGE between liver and kidney tissues in the setting of e-cigarette vapor inhalation with and without nicotine. Scatter plots comparing the log fold change (LogFC) of genes differentially expressed in liver and kidney tissues for vehicle control (VEH) and e-cigarette vapor (E-cig) exposure conditions. Linear regression was performed to assess the relationship between gene expression changes in liver and kidney tissues. Each point represents a gene with its LogFC in the kidney plotted on the x-axis and its LogFC in the liver plotted on the y-axis. The dashed horizontal and vertical lines indicate LogFC = 0, representing no change in gene expression. A linear regression line (solid black) is overlaid with a shaded 95% confidence interval. **(A)** In VEH exposed mice, regression analysis identified a strong positive correlation between LogFC in kidney and liver tissues (slope = 0.541, *p* < 0.001, R² = 0.217), indicating that genes tend to exhibit coordinated expression changes across the two tissues in the setting of chronic exposure to inhaled PG: VG without nicotine (VEH). **(B)** Regression analysis revealed a weaker and statistically non-significant correlation between LogFC in kidney and liver tissues (slope = -0.120, *p* = 0.103, R² = 0.012) of mice chronically exposed to nicotine containing e-cigarette aerosols (E-cig). This suggests more divergent gene expression patterns between liver and kidney tissues in the setting of chronic nicotine containing E-cig exposure

Scatter plots of log fold change (LogFC) values between liver and kidney tissues (Fig. 6) highlight divergent transcriptional responses across tissues and exposure conditions. With chronic no-nicotine VEH exposure (Fig. 6A), a significant positive correlation (slope = 0.541, R² = 0.217, *p* < 0.001) was observed, indicating comparable transcriptional responses in liver and kidney tissues. In contrast, in the setting of chronic exposure to nicotine containing EV (Fig. 6B), there was no correlation between liver and kidney (slope = -0.120, R² = 0.012, *p* = 0.103). These findings suggest that chronic inhalation of nicotine induces tissue-specific gene expression in the liver and kidneys.

## Discussion

The transcriptomic profile changes revealed in this study demonstrate that chronic e-cigarette exposure leads to divergent gene expression changes in liver and kidney tissues, reflecting organ-specific responses to inhaled aerosols.

In terms of the gene-level changes in the kidney, chronic e-cigarette exposure led to molecular changes highlighting the susceptibility of the renal system to fibrotic remodeling and dysfunction. *Carbonic Anhydrase 3* (*Car3*) was significantly downregulated in both E-cig and VEH groups. Because *Car3* plays a crucial role in maintaining acid-base balance and pH homeostasis [35], its suppression underscores disruption in renal buffering capacity, increasing the risk of metabolic disturbances. Similarly, downregulation of *CD36*, a key regulator of fatty acid metabolism and inflammation, suggests impaired lipid processing and heightened inflammatory responses, both of which contribute to fibrosis [36].

Reduced expression of *Heat Shock Protein Family Member DNAJB1* (*Dnaja1*), which assists in protein folding and stress response, indicates a diminished ability of kidney cells to cope with oxidative stress and misfolded protein accumulation, processes often implicated in tissue injury and fibrotic progression [37]. Conversely, genes such as *Protein Tyrosine Phosphatase*,* Receptor Type B* (*Ptprb*) and *Forkhead Box O3* (*Foxo3*) were upregulated, reflecting potential compensatory mechanisms [38]. *Ptprb* upregulation may enhance signaling pathways related to cellular proliferation [38] while *Foxo3* could support oxidative stress resistance, potentially counteracting some of the adverse effects of e-cig exposure [39].

Notably, the upregulation of *Collagen Type XXVII Alpha 1 Chain* (*Col27a1*), a major extracellular matrix component, aligns with fibrotic remodeling observed in the kidney [40]. Increased *Col27a1* expression suggests enhanced collagen deposition and extracellular matrix restructuring, hallmark features of fibrosis. These findings collectively emphasize that chronic e-cig exposure alters key pathways in the kidney, promoting inflammation, oxidative stress, and extracellular matrix remodeling, which are central to the development of fibrosis.

In terms of the gene-level changes in the liver, gene expression in liver tissues of mice exposed chronically to e-cigarette aerosols demonstrated changes particularly in pathways associated with lipid metabolism, inflammation, and oxidative stress. *Cytochrome P450 Family 8 Subfamily B Member 1* (*Cyp8b1*), upregulated in EV-exposed mice, plays a pivotal role in bile acid synthesis and cholesterol metabolism [41, 42]. Elevated *Cyp8b1* expression may disrupt bile acid homeostasis [41, 42], leading to hepatocyte injury and chronic inflammation, both of which are significant drivers of liver fibrosis. Another critical gene, *3-Hydroxy-3-Methylglutaryl-CoA Synthase 1* (*Hmgcs1*), essential for cholesterol biosynthesis, was also upregulated in EV-exposed mice [43]. Excessive cholesterol synthesis can destabilize cell membranes [43], triggering the release of inflammatory cytokines and damage-associated molecular patterns (DAMPs). These molecules activate immune responses, amplifying hepatocyte injury and fibrotic progression. Further, elevation of *Interleukin 6 Receptor Subunit Alpha* (*Il6ra*) in livers of VEH mice underscores the role of inflammatory pathways in e-cig-induced liver damage [44, 45]. Dysregulated *IL-6/STAT3* signaling has been implicated in various inflammatory and autoimmune conditions, linking e-cig exposure to chronic liver inflammation. Upregulated genes such as *Lipin 1* (*Lpin1*) and *Metallothionein 1* (*Mt1*) point to a complex interplay between lipid metabolism, oxidative stress, and inflammation [46–50]. While *Lpin1* supports lipid biosynthesis and fatty acid oxidation, its dysregulation can exacerbate metabolic imbalances. Mt1, a key regulator of metal homeostasis and oxidative stress response, suggests an adaptive attempt to mitigate cellular damage induced by chronic e-cig exposure. Conversely, the downregulation of Sterol Regulatory *Element-Binding Transcription Factor 1* (*Srebf1*), which regulates lipid homeostasis, reflects impaired lipid production, potentially contributing to metabolic disorders such as non-alcoholic fatty liver disease (NAFLD) [51, 52]. These findings underscore the multifaceted impact of e-cig exposure on the liver, revealing disruptions in lipid metabolism, inflammation, and oxidative stress pathways, all of which contribute to fibrotic disease progression.

Pathway enrichment analyses highlighted that in the kidney, non-nicotine vehicle exposures preferentially disrupted circadian rhythm, AMPK signaling, lysine degradation, FoxO signaling, and fatty acid metabolism, pathways central to renal energy balance and nutrient sensing. This suggests that even nicotine-free e-cigarette aerosols can disrupt metabolic timing, stress adaptation, and lipid handling in the kidney, predisposing to long-term renal dysfunction [26, 53]. In contrast, nicotine-containing exposures perturbed lysine degradation [54, 55], peroxisome activity, oxidative phosphorylation, focal adhesion, and protein processing in the endoplasmic reticulum, implicating nicotine in amplifying oxidative stress [56], mitochondrial injury, and extracellular matrix remodeling, processes that contribute to kidney fibrosis and loss of function. In the liver, vehicle exposures strongly enriched pathways including ferroptosis [57], insulin signaling, arginine biosynthesis, alcoholic liver disease, and NAFLD. These findings indicate that the solvents and additives in e-cigarette aerosols, independent of nicotine, can promote iron-dependent oxidative injury, disrupt amino acid and glucose metabolism, and drive disease pathways resembling both alcoholic and non-alcoholic fatty liver injury. By comparison, nicotine-containing exposures targeted steroid and terpenoid backbone biosynthesis, chemical carcinogenesis (reactive oxygen species), insulin resistance, NAFLD, and MAPK signaling. This pattern suggests that nicotine amplifies hepatic metabolic stress by perturbing cholesterol and lipid biosynthesis, enhancing ROS-mediated carcinogenic signaling, and promoting insulin resistance, all of which can accelerate progression to steatosis, inflammation, and fibrosis. These findings emphasize the systemic and organ-specific risks of e-cigarette use, reinforcing the need for further research into its long-term health impacts [58].

One limitation of this study is the use of a whole-body exposure system instead of a nose-only system. While whole-body exposure was chosen to minimize stress on the mice, which is known to occur with nose-only systems leading to altered inflammatory responses [59, 60], whole-body exposure does lead to deposition of aerosols on fur and skin and thus ingestion via grooming and transdermal absorption. Ingestion and transdermal absorption of vaping aerosol constituents may increase systemic nicotine levels and influence renal and hepatic transcriptomes, potentially amplifying downstream biological effects. Even though we minimized the time between euthanasia and organ harvest, it is possible that the time between euthanasia, harvest, and placement into liquid nitrogen may have compromised RNA integrity or led to ischemia and hypoxia related gene expression changes. Another limitation is that these data capture RNA expression at a single time point (3 months of e-cigarette aerosol inhalation), offering only a snapshot of the molecular changes occurring due to vaping. In the future, a longitudinal study would enable characterization of dynamic changes in inflammatory and fibrotic pathways over time, providing a more comprehensive understanding of e-cig-induced damage. For example, if e-cigarette aerosol inhalation induced inflammation in the acute (1–3 days) or sub-acute (1–2 weeks) phase, our data would only capture RNA expression post-inflammation resolution, thus underestimating or missing inflammatory patterns. Additionally, we did not evaluate for a dose response of nicotine in e-cig aerosols. Given nicotine’s known biological effects, future studies should explore how varying concentrations influence gene expression and fibrosis-related pathways, particularly since the latest e-cigarettes (4th generation, pod devices) use much higher concentrations than prior generations. Also, 4th generation e-cigarettes utilize nicotinic salts (slower absorption and slower more sustained nicotinic receptor engagement) instead of freebase nicotine (more lipophilic with higher mucosal absorption, and more rapid activation of nicotinic receptors), which may also alter biological effects on downstream organs. Thus, it will be important to assess the health effects of 4th generation e-devices. Further, young versus older e-cigarette vapers may have different susceptibility to effects on the renal and hepatic systems. It will be important to conduct studies in both young and older subjects in future studies to better evaluate vaping effects across the lifespan.

An important translational limitation of this study is the use of a murine model, in which the *cytochrome P450* (*CYP450*) gene repertoire and substrate specificity differ substantially from those in humans, as has been well documented for the *CYP450* superfamily. In our data, *Cyp8b1* was the only xenobiotic metabolism gene to reach statistical significance (Liver: E-cig vs. Air, logFC = + 1.37, FDR = 5.7 × 10⁻⁵), among 92 *CYP450* and related xenobiotic metabolism genes detected in our dataset. The human ortholog of mouse *Cyp8b1* is *CYP8B1* (ENSG00000180432), a high-confidence one-to-one ortholog with 75% amino acid sequence identity, providing a basis for biological plausibility, while acknowledging that substrate specificity may nonetheless differ between species. We searched publicly available transcriptomic databases (NCBI GEO and Array Express) for human or non-human primate kidney or liver tissue from e-cigarette users, but no such datasets currently exist, underscoring both the novelty and the translational gap that this study aims to address. Future studies using human-relevant in vitro models or human tissue, when ethically and practically feasible, will be necessary to confirm whether the transcriptional signals identified here translate to human biology. Despite these limitations, this study provides critical insights into the molecular effects of chronic e-cig exposure, particularly its role in promoting transcriptional changes consistent with fibrosis and metabolic dysregulation in the liver and kidneys and enhances our understanding of the systemic impact of e-cigarettes.

## Conclusions

Just as combustible cigarette smoking exerts widespread effects across cells and tissues throughout the body, e-cigarette vaping appears to produce similarly broad systemic consequences. While characterizing the cellular and molecular effects of e-cigarette vaping on the lung remains essential, given that this organ sustains the highest level of direct exposure to e-cigarette chemicals, the present findings underscore the importance of examining extrapulmonary organs to fully elucidate the potential long-term health consequences of vaping. Taken together, these data advance our mechanistic understanding of e-cigarette vaping-associated pathology and may inform both public health messaging and the development of evidence-based regulatory policy.

## Supplementary Information

Below is the link to the electronic supplementary material.

Supplementary Material 1

Supplementary Material 2

Supplementary Material 3

Supplementary Material 4

Supplementary Material 5

Supplementary Material 6

Supplementary Material 7

## Data Availability

Data will be uploaded to a publicly available data repository upon publication. Authors will also share the data upon reasonable request. Competing interests.
