# Supplementary material for: E-cigarette vaping is associated with pro-fibrotic gene expression in kidney and liver tissues
Source: J Mol Med (Berl). 2026 Jul 31;104(1):99. doi: 10.1007/s00109-026-02699-1 (PMC13424587; doi:10.1007/s00109-026-02699-1)
Supplement: Supplementary file 1 — Supplementary Material 1 [file 109_2026_2699_MOESM1_ESM.pdf]

Pathway Name and Function	Gene Abbreviation	Gene Name	E-cigarette Exposure Effect on Gene Function
**ECM and Cytoskeletal Pathway**<br><br>Production of proteins that contribute to ECM and cytoskeleton structure and function; crucial in maintaining tissue integrity and cellular function	COL1A1	Collagen Type I Alpha 1 Chain	Upregulation: ECM accumulation and fibrosis
	COL3A1	Collagen Type III Alpha 1 Chain	Upregulation: ECM accumulation and fibrosis
	COL4A1	Collagen type IV Alpha 1 Chain	Upregulation: Disruption of basal membrane structure and fibrosis
	FN1	Fibronectin	Upregulation: Fibrosis; ECM glycoprotein; Promotes cell adhesion, migration, & wound healing
	ITGA5	Integrin Alpha 5	Upregulation: Facilitates cell migration, ECM remodeling, and cell-ECM adhesion
	ITGB1	Integrin Beta 1	Upregulation: Fibrosis progression by mediating cell-ECM interactions
	MMP2	Matrix Metalloproteinase 2	Downregulation: Excessive ECM deposition due to decreased ECM degradation
	MMP9	Matrix Metalloproteinase 9	Downregulation: Excessive ECM deposition due to decreased ECM degradation
	VIM	Vimentin	Upregulation: Increased epithelial-mesenchymal transition (EMT) facilitation (key fibrosis process) as intermediate filament protein
**TGF-β Signaling Pathway**<br><br>Activation of fibroblasts and stimulating them to differentiate into myofibroblasts, which produce ECM proteins	TGFB1	Transforming Growth Factor Beta 1	Upregulation: promotes fibrosis by increasing ECM production
	TGFB2	Transforming Growth Factor Beta 2	Upregulation: promotes fibrosis by increasing ECM production
	TGFB3	Transforming Growth Factor Beta 3	Upregulation: promotes fibrosis by increasing ECM production
	TGFBR1	TGF-β receptor 1	Upregulation: Promotes fibrosis by mediating TGF -β signaling
	TGFBR2	TGF-β receptor 2	Upregulation: Promotes fibrosis by propagating TGF-β signals with TGFBR1
	SMAD2	SMAD family member 2	Upregulation: Increased ECM production and fibrosis by transducing TGF-β signals
	SMAD3	SMAD family member 3	Upregulation: Promotes fibrosis by mediating TGF-β signaling with SMAD2
	SMAD4	SMAD family member 4	Upregulation: Promotes fibrosis by mediating TGF-β signaling
	SMAD7	SMAD family member 7	Downregulation: Promotes fibrosis by inhibiting TGF-β signaling
	THBS1	Thrombospondin 1	Upregulation: Promotes fibrosis by activating TGF-β
	LTBP1	Latent TGF-β binding protein 1	Upregulation: Promotes fibrosis by regulating TGF-β availability
	FST	Follistatin	Upregulation: Promotes fibrosis by binding to and inhibiting activins and dysregulating TGF-β pathway
	BMP2	Bone morphogenetic protein 2	Upregulation: Promotes fibrosis by modulating TGF-β pathway
	BMP4	Bone morphogenetic protein 4	Upregulation: Promotes fibrosis by modulating TGF-β pathway
**Wnt/β-Catenin Signaling Pathway**<br><br>Promotes fibrosis by inducing expression of fibrogenic genes	WNT1	Wingless-Type MMTV Integration Site Family Member 1	Upregulation: Increases ECM production and fibrogenesis by increasing TGF- β signaling
	CTNNB1	B-Catenin	Upregulation: Increased expression of fibrogenic genes and ECM deposition by transducing Wnt signals
	DVL1	Dishevelled Segment Polarity Protein 1	Upregulation: Increased fibrosis by promoting Wnt signaling
	AXIN1	Axis Inhibition Protein 1	Downregulation: Promotes fibrosis by negatively regulating β-catenin
	APC	Adenomatous Polyposis Coli	Downregulation: Promotes fibrosis due to β-catenin accumulation; Tumor suppressor that regulates β-catenin
	LRP5	Low-density Lipoprotein Receptor-Related Protein 5	Upregulation: Promotes fibrosis by facilitating Wnt signaling as co-receptor
	LRP6	Low-density Lipoprotein Receptor-Related Protein 6	Upregulation: Promotes fibrosis by facilitating Wnt signaling as co-receptor
**Notch Signaling Pathway**<br><br>Involved in development of fibrosis by facilitating myofibroblast formation and epithelial-mesenchymal transition	NOTCH1	Neurogenic Locus Notch Homolog Protein 1	Upregulation: Promotes fibrosis by mediating Notch signaling as a receptor
	NOTCH2	Neurogenic Locus Notch Homolog Protein 2	Upregulation: Promotes fibrosis by mediating Notch signaling as a receptor
	NOTCH3	Neurogenic Locus Notch Homolog Protein 3	Upregulation: Promotes fibrosis by mediating Notch signaling as a receptor
	NOTCH4	Neurogenic Locus Notch Homolog Protein 4	Upregulation: Promotes fibrosis by mediating Notch signaling as a receptor
	JAG1	Jagged 1	Upregulation: Promotes fibrosis by facilitating Notch signaling as ligand for Notch receptors
	JAG2	Jagged 2	Upregulation: Promotes fibrosis by facilitating Notch signaling as ligand for Notch receptors
	DLL1	Delta-like Ligand 1	Upregulation: Promotes fibrosis by activating Notch signaling
	DLL3	Delta-like Ligand 3	Upregulation: Promotes fibrosis by activating Notch signaling
	DLL4	Delta-like Ligand 4	Upregulation: Promotes fibrosis by activating Notch signaling
	MAML1	Mastermind-like 1	Upregulation: Promotes fibrosis as coactivator in Notch signaling
	MAML2	Mastermind-like 2	Upregulation: Promotes fibrosis as coactivator in Notch signaling
	MAML3	Mastermind-like 3	Upregulation: Promotes fibrosis as coactivator in Notch signaling
	RBPJ	Recombination Signal Binding Protein for Immunoglobulin Kappa J Region	Upregulation: Promotes fibrosis by transducing Notch signals
	PIK3CA	Phosphatidylinositol-4,5-bisphosphate 3-kinase catalytic subunit alpha	Upregulation: Promotes fibrosis by activating AKT
	PIK3CB	Phosphatidylinositol-4,5-bisphosphate 3-kinase catalytic subunit beta	Upregulation: Promotes fibrosis by activating AKT
	PIK3CD	Phosphatidylinositol-4,5-bisphosphate 3-kinase catalytic subunit delta	Upregulation: Promotes fibrosis by activating AKT
	PIK3CG	Phosphatidylinositol-4,5-bisphosphate 3-kinase catalytic subunit gamma	Upregulation: Promotes fibrosis by activating AKT
	AKT1	AKT Serine/ Threonine Kinase 1	Upregulation: Promotes fibrosis by facilitating TGF- β dependent ECM production and myofibroblast differentiation

	AKT2	AKT Serine/ Threonine Kinase 2	Upregulation: Promotes fibrosis by facilitating TGF- β dependent ECM production and myofibroblast differentiation
	AKT3	AKT Serine/ Threonine Kinase 3	Upregulation: Promotes fibrosis by facilitating TGF- β dependent ECM production and myofibroblast differentiation
	PTEN	Phosphatase and Tensin Homolog	Downregulation: Promotes fibrosis due to decreased negative regulation of PI3K/AKT signaling
	PDPK1	3-Phosphoinositide Dependent Protein Kinase 1	Upregulation: Promotes fibrosis by activating AKT
	MTOR	Mechanistic Target of Rapamycin Kinase	Upregulation: Promotes fibrosis by regulating cell growth and survival
	RPS6KB1	Ribosomal Protein S6 Kinase B1	Upregulation: Promotes fibrosis by regulating cell growth and survival
**PI3K/AKT Signaling Pathway**	BAD	BCL2 Associated Agonist of Cell Death	Upregulation: Promotes fibrosis by inducing apoptosis and causing oxidative stress and inflammation
	TSC1	TSC Complex Subunit 1	Downregulation: Promotes fibrosis because of decreased negative regulation of mTOR
Signaling pathway involved in inflammation, oxidative stress, cell apoptosis, epithelial mesenchymal transformation, and autophagy	TSC2	TSC Complex Subunit 2	Downregulation: Promotes fibrosis because of decreased negative regulation of mTOR
	HRAS	Harvey Rat Sarcoma Viral Oncogene Homolog	Upregulation: Promotes cell proliferation and fibrosis via activation of MAPK signaling as proto-oncogene and GTPase
	KRAS	Kirsten Rat Sarcoma Viral Oncogene Homolog	Upregulation: Promotes cell proliferation and fibrosis via activation of MAPK signaling as proto-oncogene and GTPase
**RAS/MAPK Signaling Pathway**	NRAS	Neuroblastoma RAS Viral Oncogene Homolog	Upregulation: Promotes cell proliferation and fibrosis via activation of MAPK signaling as proto-oncogene and GTPase
	BRAF	B-Raf Proto-Oncogene, Serine Threonine Kinase	Upregulation: Promotes fibrosis via MAPK signaling activation
Pathway involved in regulation of cellular processes involved in renal fibrosis, such as activation and proliferation of myofibroblasts or extracellular matrix protein accumulation	RAF1	Raf-1-Proto-Oncogene, Serine/Threonine Kinase	Upregulation: Promotes fibrosis via MAPK signaling activation
	MAP2K1	Mitogen-Activated Protein Kinase Kinase 1	Upregulation: Promotes fibrosis via MAPK signaling activation
	MAP2K2	Mitogen-Activated Protein Kinase Kinase 2	Upregulation: Promotes fibrosis via MAPK signaling activation
	MAPK1	Mitogen-Activated Protein Kinase 1	Upregulation: Promotes fibrosis by influencing MAPK signaling downstream
	MAPK3	Mitogen-Activated Protein Kinase 3	Upregulation: Promotes fibrosis by influencing MAPK signaling downstream
	DUSP6	Dual Specificity Phosphatase 6	Downregulation: Promotes fibrosis by negatively regulating MAPK signaling
	SOS1	Son of Sevenless Homolog 1	Upregulation: Promotes fibrosis by activating RAS and amplifying MAPK signaling
	SHC1	SHC Adaptor Protein 1	Upregulation: Promotes fibrosis by mediating signaling from receptor tyrosine kinases to RAS
	NFKB1	Nuclear Factor Kappa B Subunit 1	Upregulation: Promotes kidney fibrosis by activating transcription of pro-inflammatory and fibrogenic genes and mediating NF-κB signaling as p50 subunit
	NFKB2	Nuclear Factor Kappa B Subunit 2	Upregulation: Promotes kidney fibrosis by mediating NF-κB signaling as p52 subunit
	RELA	RELA Proto-Oncogene, NFKB Subunit	Upregulation: Promotes transcription of pro-inflammatory and fibrogenic genes in kidney fibrosis and mediates NF-κB signaling as p65 subunit
	RELB	RELB Proto-Oncogene, NFKB Subunit	Upregulation: Contributes to kidney fibrosis through activation of pro-fibrotic genes and mediatation of alternative NF-κB signaling
	REL	REL Proto-Oncogene, NFKB Subunit	Upregulation: Promotes kidney fibrosis through transcription of pro-inflammatory and fibrogenic genes and mediating NF-κB signaling
	IKBKG	Inhibitor of Nuclear Factor Kappa B Kinase Regulatory Subunit Gamma	Downregulation: Promotes kidney fibrosis via NF-κB activation
**NF-κB Signaling Pathway**	IKBKB	Inhibitor of Nuclear Factor Kappa B Kinase Regulatory Subunit Beta	Upregulation: Promotes transcription of pro-inflammatory and fibrgenic genes in kidney fibrosis via NF-κB signaling activation
	IKBKA	Inhibitor of Nuclear Factor Kappa B Kinase Regulatory Subunit Alpha	Upregulation: Promotes kidney fibrosis by activating NF-κB signaling
Signaling pathway known to promote renal fibrosis by causing hyperactivation of NFKB, triggering recruitment and activation of immune cells, resulting in inflammation and oxidative stress.	NFKBIA	NFKB Inhibitor Alpha	Downregulation: Promotes kidney fibrosis via NF-κB activation
	NFKBIB	NFKB Inhibitor Beta	Downregulation: Promotes kidney fibrosis via NF-κB signaling regulation
	TNFAIP3	Tumor Necrosis Factor Alpha-Induced Protein 3	Downregulation: Promotes kidney fibrosis via NF-κB activation
	BCL3	B-cell Lymphoma 3 Protein	Upregulation: Promotes kidney fibrosis via regulation of NF-κB signaling and transcription of specific NF-κB target genes
	TRAF1	TNF Receptor-Associated Factor 1	Upregulation: Involved in kidney fibrosis through activating NF-κB and mediating NF-κB signaling
	TRAF2	TNF Receptor-Associated Factor 2	Upregulation: Involved in kidney fibrosis through activating NF-κB and mediating NF-κB signaling
	TRAF6	TNF Receptor-Associated Factor 6	Upregulation: Involved in kidney fibrosis through activating NF-κB and mediating NF-κB signaling
	LTA	Lymphotoxin Alpha	Upregulation: Promotes kidney fibrosis via NF-κB signaling activation and induction of inflammatory and fibrogenic responses
	LTB	Lymphotoxin Beta	Upregulation: Promtoes kidney fibrosis via NF-κB signaling activation
	TNF	Tumor Necrosis Factor	Upregulation: induces kidney fibrosis via NF-κB signaling activation, causing inflammation and ECM remodeling
	IL1A	Interleukin 1 Alpha	Upregulation: promotes kidney fibrosis via NF-κB signaling activation, resulting in inflammatory responses
	IL1B	Interleukin 1 Beta	Upregulation: Promotes kidney fibrosis via NF-κB signaling activation

	IL6	Interleukin-6	Upregulation: Promotes kidney fibrosis via NF-κB signaling activation, resulting in inflammation and fibrogenic responses
**JAK/STAT Signaling Pathway**<br><br>Signaling pathway that mediates various biological processes such as cell proliferation, differentiation, apoptosis, and immune responses, due to which, prolonged activation can promote fibrosis and inflammation.	JAK1	Janus Kinase 1	Upregulation: Promotes kidney fibrosis by activating transcription of fibrogenic genes
	JAK2	Janus Kinase 2	Upregulation: Promotes kidney fibrosis by activating transcription of fibrogenic genes
	JAK3	Janus Kinase 3	Upregulation: Promotes kidney fibrosis by activating transcription of fibrogenic genes
	TYK2	Tyrosine Kinase 2	Upregulation: Promotes kidney fibrosis by activating transcription of fibrogenic genes
	STAT1	Signal Transducer and Activator of Transcription 1	Upregulation: Promotes kidney fibrosis by activating transcription of fibrogenic genes
	STAT2	Signal Transducer and Activator of Transcription 2	Upregulation: Promotes kidney fibrosis by activating transcription of fibrogenic genes
	STAT3	Signal Transducer and Activator of Transcription 3	Upregulation: Promotes kidney fibrosis by activating transcription of fibrogenic genes
	STAT4	Signal Transducer and Activator of Transcription 4	Upregulation: Promotes kidney fibrosis by activating transcription of fibrogenic genes
	STAT5A	Signal Transducer and Activator of Transcription 5A	Upregulation: Promotes kidney fibrosis by activating transcription of fibrogenic genes
	STAT5B	Signal Transducer and Activator of Transcription 5B	Upregulation: Promotes kidney fibrosis by activating transcription of fibrogenic genes
	STAT6	Signal Transducer and Activator of Transcription 6	Upregulation: Promotes kidney fibrosis by activating transcription of fibrogenic genes
	SOCS1	Suppressor of Cytokine Signaling 1	Downregulation: Negatively regulates JAK/STAT signaling; promotes JAK/STAT activation and kidney fibrosis
	SOCS3	Suppressor of Cytokine Signaling 3	Downregulation: Negatively regulates JAK/STAT signaling; promotes JAK/STAT activation and kidney fibrosis
	SH2B1	SH2B Adaptor Protein 1	Upregulation: Facilitates JAK/STAT activation and transcription of fibrogenic genes in kidney fibrosis
**CDK Fibrosis Pathway**<br><br>Signaling pathway that plays a role in regulation of cell division and driving cell division.	TGFBR1	Transforming Growth Factor Beta Receptor 1	Upregulation: promotes kidney fibrosis by activating SMAD proteins and initiating TGF-β signaling
	TGFBR2	Transforming Growth Factor Beta Receptor 2	Upregulation: promotes kidney fibrosis by activating SMAD proteins and initiating TGF-β signaling
	SMAD2	Mothers Against Decapentaplegic Homolog 2	Upregulation: Promotes kidney fibrosis by activating transcription of fibrogenic genes and mediating TGF-β signals
	SMAD3	Mothers Against Decapentaplegic Homolog 3	Upregulation: Promotes kidney fibrosis by activating transcription of fibrogenic genes and mediating TGF-β signals
	COL1A1	Collagen Type 1 Alpha 1 Chain	Upregulation: Promotes kidney fibrosis by increasing collagen production and ECM deposition
	COL1A2	Collagen Type 1 Alpha 2 Chain	Upregulation: Promotes kidney fibrosis by increasing collagen production and ECM deposition
	COL3A1	Collagen Type 3 Alpha 1 Chain	Upregulation: Promotes kidney fibrosis by increasing collagen production and ECM deposition
	COL5A2	Collagen Type 5 Alpha 2 Chain	Upregulation: Promotes kidney fibrosis by increasing collagen production and ECM deposition
	COL7A1	Collagen Type 7 Alpha 1 Chain	Upregulation: Promotes kidney fibrosis by increasing collagen production and ECM deposition
	PDGFRB	Platelet-Derived Growth Factor Receptor Beta	Upregulation: Promotes kidney fibrosis by increasing cell proliferation, enhancing myofibroblast activation, and ECM production
	ACTA2	Actin Alpha 2	Upregulation: Contributes to kidney fibrosis by inducing ECM contraction
	TAGLN	Transgelin	Upregulation: Facilitates myofibroblast differentiation and ECM production in kidney fibrosis
