# Supplementary material for: E-cigarette vaping is associated with pro-fibrotic gene expression in kidney and liver tissues
Source: J Mol Med (Berl). 2026 Jul 31;104(1):99. doi: 10.1007/s00109-026-02699-1 (PMC13424587; doi:10.1007/s00109-026-02699-1)
Supplement: Supplementary file 2 — Supplementary Material 2 [file 109_2026_2699_MOESM2_ESM.pdf]

Pathway Name and Function	Gene Abbreviation	Gene Name	E-cigarette Exposure Effect on Gene Function
**Adipocytokine Signaling Pathway**<br>Hormones released from adipocytokines associated with obesity and metabolism issues, promoting inflammation and fibrosis	ADIPOQ	Adiponectin	Downregulation: increased inflammation and liver fibrosis
	LEP	Leptin	Upregulation: increased hepatic inflammation and fibrosis
	TNF	Tumor Necrosis Factor	Upregulation: Promotes liver cell damage and fibrosis
	IL6	Interleukin 6	Upregulation: Induces inflammatory response, resulting in chronic liver inflammation and fibrosis
	PPARγ	Peroxisome Proliferator-Activated Receptor Gamma	Downregulation: Increased inflammation and liver fibrosis
	AMPK	AMP-Activated Protein Kinase	Downregulation: Increased lipid accumulation and liver fibrosis
**AMPK Signaling Pathway**<br>Promotes hepatic fibrosis and inflammation via excessive ECM deposition and collagen accumulation	LKB1	Liver Kinase B1	Downregulation: Exacerbated inflammation and liver fibrosis
	ACC	Acetyl-CoA Carboxylase	Upregulation: Increased lipid accumulation and liver fibrosis
	PGC1α	Peroxisome Proliferator-Activated Receptor Gamma Coactivator 1-alpha	Downregulation: Mitochondrial dysfunction and liver fibrosis
**Apoptosis Signaling Pathway**<br>Promotes hepatocyte apoptosis via phagocytosis and receptor mediation, contributing to liver fibrosis	CASP3	Caspase-3	Upregulation: Promotes apoptosis, contributing to liver fibrosis
	CASP8	Caspase-8	Upregulation: Promotes apoptosis, contributing to liver fibrosis
	CASP9	Caspase-9	Upregulation: Promotes apoptosis, contributing to liver fibrosis
	BAX	Bcl-2-associated X protein	Upregulation: Promotes apoptosis, contributing to liver fibrosis
	BAK	Bcl-2 homologous antagonist/killer	Upregulation: Promotes apoptosis, contributing to liver fibrosis
	BCL2	B-cell lymphoma 2	Downregulation: Promotes apoptosis, contributing to liver fibrosis
	FAS	Fas Cell Surface Death Receptor	Upregulation: Promotes apoptosis, contributing to liver fibrosis
	FADD	Fas-Associated Death Domain Protein	Upregulation: Promotes apoptosis, contributing to liver fibrosis
**Arginine and Proline Metabolism Pathway**<br>Arginine catabolic enzymes lead to hepatic stellate cell (HSC) activation, which increases ECM deposition, and contributes to liver fibrosis and inflammation.	ARG1	Arginase 1	Downregulation: Dysregulation of urea cycle, contributing to liver fibrosis
	NOS2	Nitric Oxide Synthase 2	Upregulation: Promotes oxidative stress, inducing liver fibrosis
	NOS3	Nitric Oxide Synthase 3	Downregulation: Contributes to vascular dysfunction, inducing liver fibrosis
**Autophagy Pathway**<br>Increased autophagy activates HSCs, leading to ECM accumulation and degradation, promoting inflammation and liver fibrosis	BECLIN1	Beclin 1	Downregulation: Impaired autophagy, leading to liver fibrosis
	SQSTM1	Sequestosome 1	Upregulation: p62 accumulation impairs autophagy, leading to liver fibrosis
**Base Excision Repair Pathway**<br>Improper repair of oxidative DNA damage leads to inflammation and liver fibrosis	OGG1	8-Oxoguanine DNA Glycosylase	Downregulation: Increased DNA damage, contributing to liver fibrosis
	APEX1	AP Endonuclease 1	Downregulation: Increased DNA damage, contributing to liver fibrosis
	POLB	DNA Polymerase Beta	Downregulation: Impaired DNA repair, contributing to liver fibrosis
	LIG3	DNA Ligase III	Downregulation: Increased DNA damage, contributing to liver fibrosis
**Bile Acid Signaling Pathway**<br>Altered bile acid concentrations signal and initiate inflammatory responses, contributing to liver fibrosis	FXR	Farnesoid X Receptor	Downregulation: Dysregulated bile acid metabolism and liver fibrosis
	SHP	Small Heterodimer Partner	Downregulation: Dysregulated bile acid metabolism and liver fibrosis
	CYP7A1	Cholesterol 7 alpha-Hydroxylase	Downregulation: Bile acid synthesis inhibition, bile acid metabolism dysregulation, and liver fibrosis
	BSEP	Bile Salt Export Pump	Downregulation: Bile acid transport inhibition, cholestasis, and liver fibrosis
	NTCP	Sodium Taurocholate Co-Transporting Polypeptide	Downregulation: Bile acid transport inhibition, cholestasis, and liver fibrosis
**Chemokine Signaling Pathway**<br>Chemokine and their receptors trigger activation and accumulation of immune cells, leading to inflammation and liver fibrosis	CCL2	C-C Motif Chemokine Ligand 2	Upregulation: Induces inflammatory response, promoting liver fibrosis
	CCL5	C-C Motif Chemokine Ligand 5	Upregulation: Induces inflammatory response, promoting liver fibrosis
	CXCL8	C-X-C Motif Chemokine Ligand 8	Upregulation: Induces inflammatory response, promoting liver fibrosis
	CCR1	C-C Motif Chemokine Receptor 1	Upregulation: Induces inflammatory response, promoting liver fibrosis
	CCR2	C-C Motif Chemokine Receptor 2	Upregulation: Induces inflammatory response, promoting liver fibrosis
	CCR5	C-C Motif Chemokine Receptor 5	Upregulation: Induces inflammatory response, promoting liver fibrosis
	CXCR1	C-X-C Motif Chemokine Receptor 1	Upregulation: Induces inflammatory response, promoting liver fibrosis
	CXCR2	C-X-C Motif Chemokine Receptor 2	Upregulation: Induces inflammatory response, promoting liver fibrosis
**Cholesterol Metabolism Pathway**<br>Altered cholesterol metabolism due to cholestasis and accumulation of statins promotes liver fibrosis	HMGCR	3-Hydroxy-3-Methylglutaryl-CoA Reductase	Downregulation: Cholesterol synthesis inhibition, dysregulated cholesterol metabolism, leading to liver fibrosis
	LDLR	Low-Density Lipoprotein Receptor	Downregulation: Cholesterol synthesis inhibition, dysregulated cholesterol metabolism, leading to liver fibrosis
	SCAP	SREBP Cleavage-Activating Protein	Downregulation: Cholesterol synthesis inhibition, dysregulated cholesterol metabolism, leading to liver fibrosis
	SREBP	Sterol Regulatory Element-Binding Protein	Downregulation: Cholesterol synthesis inhibition, dysregulated cholesterol metabolism, leading to liver fibrosis
**Cytokine-Cytokine Receptor Interaction Pathway**<br>Hepatocellular damage and inflammation trigger cytokine accumulation, triggering ECM deposition and liver fibrosis	IL1B	Interleukin 1 Beta	Upregulation: Induces inflammatory response, promoting inflammation and liver fibrosis
	IL6	Interleukin 6	Upregulation: Induces inflammatory response, promoting inflammation and liver fibrosis
	IL10	Interleukin 10	Upregulation: Induces inflammatory response, promoting inflammation and liver fibrosis
	IL13	Interleukin 13	Upregulation: Induces inflammatory response, promoting inflammation and liver fibrosis
	TNF	Tumor Necrosis Factor	Upregulation: Induces inflammatory response, promoting inflammation and liver fibrosis
	TNFRSF1A	TNF Receptor Superfamily Member 1A	Upregulation: Induces inflammatory response, promoting inflammation and liver fibrosis
	IL1R1	Interleukin 1 Receptor Type 1	Upregulation: Induces inflammatory response, promoting inflammation and liver fibrosis
	IL6R	Interleukin 6 Receptor	Upregulation: Induces inflammatory response, promoting inflammation and liver fibrosis
**CGAS-STING Pathway**<br>Recognizes double stranded DNA errors and activates innate immune responses and induces interferon expression, contributing to lnflammation and liver fibrosis	CGAS	Cyclic GMP-AMP Synthase	Upregulation: Induces immune response, promoting inflammation and liver fibrosis
	STING	Stimulator of Interferon Genes	Upregulation: Induces immune response, promoting inflammation and liver fibrosis
	TBK1	TANK-Binding Kinase 1	Upregulation: Induces immune response, promoting inflammation and liver fibrosis
	IRF3	Interferon Regulatory Factor 3	Upregulation: Induces immune response, promoting inflammation and liver fibrosis
**Endoplasmic Reticulum (ER) Stress Pathway**	XBP1	X-Box Binding Protein 1	Upregulation: Induces endoplasmic reticulum stress, promoting inflammation and liver fibrosis
	ATF6	Activating Transcription Factor 6	Upregulation: Induces endoplasmic reticulum stress, promoting inflammation and liver fibrosis
	PERK	Protein Kinase RNA-Like Endoplasmic Reticulum Kinase	Upregulation: Induces endoplasmic reticulum stress, promoting inflammation and liver fibrosis
	EIF2α	Eukaryotic Translation Initiation Factor 2 Alpha	Upregulation: Induces endoplasmic reticulum stress, promoting inflammation and liver fibrosis

Accumulation of misfolded or unfolded proteins leads to ER stress, promoting apoptosis and contributing to inflammation and liver fibrosis	GRP78	Glucose-Regulated Protein 78	Upregulation: Induces endoplasmic reticulum stress, promoting inflammation and liver fibrosis
**Epithelial-Mesenchymal Transition (EMT) Pathway** Production of proteins that contribute to ECM and cytoskeleton structure and function; crucial in maintaining tissue integrity and cellular function	SNAI1	Snail Family Transcriptional Repressor 1	Upregulation: Induces epithelial-mesenchymal transition, promoting liver fibrosis
	SNAI2	Snail Family Transcriptional Repressor 2	Upregulation: Induces epithelial-mesenchymal transition, promoting liver fibrosis
	TWIST1	Twist Family BHLH Transcription Factor 1	Upregulation: Induces epithelial-mesenchymal transition, promoting liver fibrosis
	ZEB1	Zinc Finger E-Box Binding Homeobox	Upregulation: Induces epithelial-mesenchymal transition, promoting liver fibrosis
	ZEB2	Zinc Finger E-Box Binding Homeobox	Upregulation: Induces epithelial-mesenchymal transition, promoting liver fibrosis
	VIM	Vimentin	Upregulation: Induces epithelial-mesenchymal transition, promoting liver fibrosis
	FN1	Fibronectin 1	Upregulation: Induces extracellular matrix remodeling, promoting liver fibrosis
**ERK5 Signaling Pathway** ECM deposition activates hepatic myofibroblasts, leading to inflammatory responses and liver fibrosis	MAPK7	Mitogen-Activated Protein Kinase 7	Upregulation: Increased oxidative stress and inflammation leads to activation of cellular stress responses and fibrogenic processes, promoting liver fibrosis
	MEKK2	Mitogen-activated protein kinase kinase kinase 2	Upregulation: Increased oxidative stress and inflammation leads to activation of cellular stress responses and fibrogenic processes, promoting liver fibrosis
	MEKK3	Mitogen-activated protein kinase kinase kinase 3	Upregulation: Increased oxidative stress and inflammation leads to activation of cellular stress responses and fibrogenic processes, promoting liver fibrosis
	MEK5	Mitogen-activated protein kinase kinase 5	Upregulation: Increased oxidative stress and inflammation leads to activation of cellular stress responses and fibrogenic processes, promoting liver fibrosis
**Extracellular Matrix (ECM) Receptor Interaction Pathway** ECM remodeling and deposition increases myofibroblasts, cytokines, and reactive oxygen species, leading to inflammation and liver fibrosis	COL1A1	Collagen, type I, alpha 1 chain	Upregulation: Increased reactive oxygen species (ROS) production and inflammatory cytokines, resulting in excessive extracellular matrix (ECM) deposition, promoting liver fibrosis
	COL1A2	Collagen type I alpha 2 chain	Upregulation: Increased reactive oxygen species (ROS) production and inflammatory cytokines, resulting in excessive extracellular matrix (ECM) deposition, promoting liver fibrosis
	COL4A1	Collagen type IV alpha 1 chain	Upregulation: Increased reactive oxygen species (ROS) production and inflammatory cytokines, resulting in excessive extracellular matrix (ECM) deposition, promoting liver fibrosis
	LAMA1	Laminin subunit alpha 1	Upregulation: Increased reactive oxygen species (ROS) production and inflammatory cytokines, resulting in excessive extracellular matrix (ECM) deposition, promoting liver fibrosis
	LAMB1	Laminin subunit beta 1	Upregulation: Increased reactive oxygen species (ROS) production and inflammatory cytokines, resulting in excessive extracellular matrix (ECM) deposition, promoting liver fibrosis
	ITGA4	Integrin subunit alpha 4	Upregulation: Increased reactive oxygen species (ROS) production and inflammatory cytokines, resulting in excessive extracellular matrix (ECM) deposition, promoting liver fibrosis
**G-Protein Coupled Receptor (GPCR) Signaling Pathway** Regulation of liver metabolism and fibrosis via hepatic stellate cell (HSC) activation	GNAS	Guanine nucleotide-binding protein G(s) subunit alpha	Downregulation: Disruption in G-protein coupled receptor signaling causes imbalance in cyclic AMP and phospholipase C pathways, resulting in disturbances in cell proliferation and apoptosis, contributing to liver fibrosis
	GNAI1	G protein subunit alpha i1	Downregulation: Disruption in G-protein coupled receptor signaling causes imbalance in cyclic AMP and phospholipase C pathways, resulting in disturbances in cell proliferation and apoptosis, contributing to liver fibrosis
	GNAQ	Guanine nucleotide-binding protein G(q) subunit alpha	Downregulation: Disruption in G-protein coupled receptor signaling causes imbalance in cyclic AMP and phospholipase C pathways, resulting in disturbances in cell proliferation and apoptosis, contributing to liver fibrosis
	GNA11	Guanine nucleotide-binding protein subunit alpha-11	Downregulation: Disruption in G-protein coupled receptor signaling causes imbalance in cyclic AMP and phospholipase C pathways, resulting in disturbances in cell proliferation and apoptosis, contributing to liver fibrosis
	ADCY	Adenylate cyclase	Downregulation: Disruption in G-protein coupled receptor signaling causes imbalance in cyclic AMP and phospholipase C pathways, resulting in disturbances in cell proliferation and apoptosis, contributing to liver fibrosis
	PLCβ	Phospholipase C Beta	Downregulation: Disruption in G-protein coupled receptor signaling causes imbalance in cyclic AMP and phospholipase C pathways, resulting in disturbances in cell proliferation and apoptosis, contributing to liver fibrosis
**Gluconeogenesis Pathway** Dysregulation of hepatic gluconeogenesis disrupts hormone release, gene transcription, and post-translational modifications, promoting inflammation and fibrosis	PCK1	phosphoenolpyruvate carboxykinase 1	Downregulation: Impaired gluconeogenic enzymes disrupts glucose metabolism and hepatic steatosis, promoting liver fibrosis
	G6PC	Glucose-6-phosphatase catalytic subunit 1	Downregulation: Impaired gluconeogenic enzymes disrupts glucose metabolism and hepatic steatosis, promoting liver fibrosis
	FBP1	Fructose-1,6-bisphosphatase 1	Downregulation: Impaired gluconeogenic enzymes disrupts glucose metabolism and hepatic steatosis, promoting liver fibrosis
	PEPCK	Phosphoenolpyruvate carboxykinase	Downregulation: Impaired gluconeogenic enzymes disrupts glucose metabolism and hepatic steatosis, promoting liver fibrosis
**Glutathione Metabolism Pathway** Increased reactive oxygen species promote cellular stress and hepatic inflammation, leading to fibrosis	GSS	Glutathione Synthetase	Downregulation: Disrupted glutathione synthesis reduces antioxidant capacity, leading to increased oxidative stress and hepatocellular injury, promoting fibrosis
	GSR	Glutathione Reductase	Downregulation: Disrupted glutathione synthesis reduces antioxidant capacity, leading to increased oxidative stress and hepatocellular injury, promoting fibrosis
	GCLM	Glutamate-cysteine ligase modifier subunit	Downregulation: Disrupted glutathione synthesis reduces antioxidant capacity, leading to increased oxidative stress and hepatocellular injury, promoting fibrosis
	GCLC	Glutamate-cysteine ligase catalytic	Downregulation: Disrupted glutathione synthesis reduces antioxidant capacity, leading to increased oxidative stress and hepatocellular injury, promoting fibrosis
**Hedgehog Signaling Pathway** Impaired liver regeneration activates hepatic stellate cells (HSCs) and promotes inflammation and liver fibrosis	SHH	Sonic hedgehog protein	Upregulation: Activation of myofibroblasts and increased ECM production promotes liver fibrosis
	PTCH1	Patched 1	Upregulation: Activation of myofibroblasts and increased ECM production promotes liver fibrosis
	SMO	Smoothened	Upregulation: Activation of myofibroblasts and increased ECM production promotes liver fibrosis
	GLI1	GLI family zinc finger 1	Upregulation: Activation of myofibroblasts and increased ECM production promotes liver fibrosis
	GLI2	GLI family zinc finger 2	Upregulation: Activation of myofibroblasts and increased ECM production promotes liver fibrosis
	GLI3	GLI family zinc finger 3	Upregulation: Activation of myofibroblasts and increased ECM production promotes liver fibrosis
	SUFU	Suppressor of fused homolog	Upregulation: Activation of myofibroblasts and increased ECM production promotes liver fibrosis
	HHIP	Hedgehog-interacting protein	Upregulation: Activation of myofibroblasts and increased ECM production promotes liver fibrosis
**Histidine Metabolism Pathway**	HAL	Histidine ammonia-lyase	Upregulation: Increased histamine production due to activation of histidine synthesis enzymes causes inflammation and liver fibrosis
	HAL2	histidine ammonia-lyase 2	Upregulation: Increased histamine production due to activation of histidine synthesis enzymes causes inflammation and liver fibrosis

Dysregulated histidine metabolism promotes immune evasion and tumor growth, promoting inflammation and liver fibrosis	HAL3	histidine ammonia-lyase 3	Upregulation: Increased histamine production due to activation of histidine synthesis enzymes causes inflammation and liver fibrosis
	HNMT	Histamine N-methyltransferase	Upregulation: Increased histamine production due to activation of histidine synthesis enzymes causes inflammation and liver fibrosis
**Integrin Signaling Pathway** Activation, differentiation, and proliferation of HSCs and ECM-secreting myofibroblasts, contributing to liver inflammation and fibrosis	ITGA1	Integrin subunit alpha 1	Upregulation: Increased integrin and focal adhesion components enhance cell adhesion and migration, increasing myofibroblast activity and ECM deposition, promoting liver fibrosis
	ITGA5	Integrin subunit alpha 5	Upregulation: Increased integrin and focal adhesion components enhance cell adhesion and migration, increasing myofibroblast activity and ECM deposition, promoting liver fibrosis
	ITGB1	Integrin beta-1	Upregulation: Increased integrin and focal adhesion components enhance cell adhesion and migration, increasing myofibroblast activity and ECM deposition, promoting liver fibrosis
	ITGB3	Integrin beta-3	Upregulation: Increased integrin and focal adhesion components enhance cell adhesion and migration, increasing myofibroblast activity and ECM deposition, promoting liver fibrosis
	TALIN	Talin-1	Upregulation: Increased integrin and focal adhesion components enhance cell adhesion and migration, increasing myofibroblast activity and ECM deposition, promoting liver fibrosis
	KINDLIN	Kindlin	Upregulation: Increased integrin and focal adhesion components enhance cell adhesion and migration, increasing myofibroblast activity and ECM deposition, promoting liver fibrosis
	FAK	Focal Adhesion Kinase	Upregulation: Increased integrin and focal adhesion components enhance cell adhesion and migration, increasing myofibroblast activity and ECM deposition, promoting liver fibrosis
	SRC	SRC proto-oncogene, non-receptor tyrosine kinase	Upregulation: Increased integrin and focal adhesion components enhance cell adhesion and migration, increasing myofibroblast activity and ECM deposition, promoting liver fibrosis
**JAK/STAT Signaling Pathway** Signaling pathway that mediates various biological processes such as cell proliferation, differentiation, apoptosis, and immune responses, due to which, prolonged activation can promote fibrosis and inflammation.	JAK1	Janus kinase 1	Upregulation: Increased production of cytokines and growth factors, resulting in inflammation and liver fibrosis
	JAK2	Janus kinase 2	Upregulation: Increased production of cytokines and growth factors, resulting in inflammation and liver fibrosis
	JAK3	Janus kinase 3	Upregulation: Increased production of cytokines and growth factors, resulting in inflammation and liver fibrosis
	TYK2	Tyrosine kinase 2	Upregulation: Increased production of cytokines and growth factors, resulting in inflammation and liver fibrosis
	STAT1	Signal Transducer and Activator of Transcription 1	Upregulation: Increased production of cytokines and growth factors, resulting in inflammation and liver fibrosis
	STAT3	Signal Transducer and Activator of Transcription 3	Upregulation: Increased production of cytokines and growth factors, resulting in inflammation and liver fibrosis
	STAT5	Signal Transducer and Activator of Transcription 5	Upregulation: Increased production of cytokines and growth factors, resulting in inflammation and liver fibrosis
	SOCS1	Suppressor of cytokine signaling 1	Upregulation: Increased production of cytokines and growth factors, resulting in inflammation and liver fibrosis
	SOCS3	Suppressor of cytokine signaling 3	Upregulation: Increased production of cytokines and growth factors, resulting in inflammation and liver fibrosis
**JNK Signaling Pathway** Dysregulation of cell differentiation, proliferation, and ROS accumulation contributes to liver inflammation and fibrosis	MAPK8	Mitogen-Activated Protein Kinase 8	Upregulation: Increased oxidative stress and inflammatory cytokines activates cellular stress responses, promoting hepatocellular injury and liver fibrosis
	MAPK9	Mitogen-Activated Protein Kinase 9	Upregulation: Increased oxidative stress and inflammatory cytokines activates cellular stress responses, promoting hepatocellular injury and liver fibrosis
	MAPK10	Mitogen-Activated Protein Kinase 10	Upregulation: Increased oxidative stress and inflammatory cytokines activates cellular stress responses, promoting hepatocellular injury and liver fibrosis
	MKK4	Mitogen-activated protein kinase kinase 4	Upregulation: Increased oxidative stress and inflammatory cytokines activates cellular stress responses, promoting hepatocellular injury and liver fibrosis
	MKK7	Mitogen-activated protein kinase kinase 7	Upregulation: Increased oxidative stress and inflammatory cytokines activates cellular stress responses, promoting hepatocellular injury and liver fibrosis
**MAPK/ERK Signaling Pathway** ECM degradation due to dysregulated cell differentiation, proliferation, and apoptosis, contributing to liver inflammation and fibrosis	MAPK1	Mitogen-activated protein kinase 1	Upregulation: Increased ROS and inflammatory cytokines enhance cell proliferation and cell survival, promoting liver fibrosis
	MAPK3	Mitogen-activated protein kinase 3	Upregulation: Increased ROS and inflammatory cytokines enhance cell proliferation and cell survival, promoting liver fibrosis
	RAF1	RAF-1 proto-oncogene, serine/threonine kinase	Upregulation: Increased ROS and inflammatory cytokines enhance cell proliferation and cell survival, promoting liver fibrosis
	MEK1	Mitogen-activated protein kinase kinase 1	Upregulation: Increased ROS and inflammatory cytokines enhance cell proliferation and cell survival, promoting liver fibrosis
	MEK2	Mitogen-activated protein kinase kinase 2	Upregulation: Increased ROS and inflammatory cytokines enhance cell proliferation and cell survival, promoting liver fibrosis
	RAS	Rat sarcoma viral oncogene homolog	Upregulation: Increased ROS and inflammatory cytokines enhance cell proliferation and cell survival, promoting liver fibrosis
	BRAF	B-Raf proto-oncogene, serine/threonine kinase	Upregulation: Increased ROS and inflammatory cytokines enhance cell proliferation and cell survival, promoting liver fibrosis
	ERK1/2	Extracellular signal-regulated kinase	Upregulation: Increased ROS and inflammatory cytokines enhance cell proliferation and cell survival, promoting liver fibrosis
**MAPK/JNK Signaling Pathway** Dysregulated autophagy and ROS accumulation lead to liver inflammation and fibrosis	MAPK8	Mitogen-activated protein kinase 8	Upregulation: Increased oxidative stress and inflammatory cytokines cause cellular stress responses, resulting in hepatocellular injury and liver fibrosis
	MAPK9	Mitogen-activated protein kinase 9	Upregulation: Increased oxidative stress and inflammatory cytokines cause cellular stress responses, resulting in hepatocellular injury and liver fibrosis
	MAPK10	Mitogen-activated protein kinase 10	Upregulation: Increased oxidative stress and inflammatory cytokines cause cellular stress responses, resulting in hepatocellular injury and liver fibrosis
	JNK1	c-Jun N-terminal kinase 1	Upregulation: Increased oxidative stress and inflammatory cytokines cause cellular stress responses, resulting in hepatocellular injury and liver fibrosis
	JNK2	c-Jun N-terminal kinase 2	Upregulation: Increased oxidative stress and inflammatory cytokines cause cellular stress responses, resulting in hepatocellular injury and liver fibrosis
	MKK4	Mitogen-activated protein kinase kinase 4	Upregulation: Increased oxidative stress and inflammatory cytokines cause cellular stress responses, resulting in hepatocellular injury and liver fibrosis
	MKK7	Mitogen-activated protein kinase kinase 7	Upregulation: Increased oxidative stress and inflammatory cytokines cause cellular stress responses, resulting in hepatocellular injury and liver fibrosis
**MTOR Signaling Pathway** Suppresses liver lipogenesis and increases fatty acid oxidation, leading to free fatty acid accumulation, promoting liver inflammation and fibrosis	mTOR	Mechanistic target of rapamycin kinase	Upregulation: Increased oxidative stress and nutrient signaling promote cell growth and survival, contributing to liver fibrosis
	RICTOR	Rapamycin-insensitive companion of mTOR	Upregulation: Increased oxidative stress and nutrient signaling promote cell growth and survival, contributing to liver fibrosis
	RAPTOR	Regulatory-associated protein of mTOR	Upregulation: Increased oxidative stress and nutrient signaling promote cell growth and survival, contributing to liver fibrosis
	AKT	AKT serine/threonine kinase	Upregulation: Increased oxidative stress and nutrient signaling promote cell growth and survival, contributing to liver fibrosis
**NF-kB Signaling Pathway**	RELA	RELA proto-oncogene, NF-kB subunit	Upregulation: Increased inflammatory cytokines and oxidative stress lead to inflammation and hepatocellular damage, promoting liver fibrosis
	RELB	RELB proto-oncogene, NF-kB subunit	Upregulation: Increased inflammatory cytokines and oxidative stress lead to inflammation and hepatocellular damage, promoting liver fibrosis

Signaling pathway known to promote fibrosis by causing hyperactivation of NFKB, triggering recruitment and activation of immune cells, resulting in inflammation and oxidative stress.	NFKB1	Nuclear factor kappa B subunit 1	Upregulation: Increased inflammatory cytokines and oxidative stress lead to inflammation and hepatocellular damage, promoting liver fibrosis
	NFKB2	nuclear factor kappa B subunit 2	Upregulation: Increased inflammatory cytokines and oxidative stress lead to inflammation and hepatocellular damage, promoting liver fibrosis
	IKBKB	Inhibitor of nuclear factor kappa B kinase subunit beta	Upregulation: Increased inflammatory cytokines and oxidative stress lead to inflammation and hepatocellular damage, promoting liver fibrosis
	IKBKG	Inhibitor of nuclear factor kappa-B kinase regulatory subunit gamma	Upregulation: Increased inflammatory cytokines and oxidative stress lead to inflammation and hepatocellular damage, promoting liver fibrosis
	IKBKA	CHUK - component of inhibitor of nuclear factor kappa B kinase complex	Upregulation: Increased inflammatory cytokines and oxidative stress lead to inflammation and hepatocellular damage, promoting liver fibrosis
	TNFAIP3	Tumor Necrosis Factor Alpha-Induced Protein 3	Upregulation: Increased inflammatory cytokines and oxidative stress lead to inflammation and hepatocellular damage, promoting liver fibrosis
**Notch Signaling Pathway**<br><br>Involved in development of fibrosis by facilitating myofibroblast formation and epithelial-mesenchymal transition	NOTCH1	Notch receptor 1	Upregulation: Enhanced cell proliferation and survival, resulting in liver fibrosis
	NOTCH2	Notch receptor 2	Upregulation: Enhanced cell proliferation and survival, resulting in liver fibrosis
	NOTCH3	Notch receptor 3	Upregulation: Enhanced cell proliferation and survival, resulting in liver fibrosis
	NOTCH4	Notch receptor 4	Upregulation: Enhanced cell proliferation and survival, resulting in liver fibrosis
	JAG1	Jagged Canonical Notch Ligand 1	Upregulation: Enhanced cell proliferation and survival, resulting in liver fibrosis
	DLL1	Delta like canonical Notch ligand 1	Upregulation: Enhanced cell proliferation and survival, resulting in liver fibrosis
	DLL4	Delta like canonical Notch ligand 4	Upregulation: Enhanced cell proliferation and survival, resulting in liver fibrosis
	HES1	Hairy and Enhancer of Split 1	Upregulation: Enhanced cell proliferation and survival, resulting in liver fibrosis
	HEY1	Hairy/enhancer-of-split related with YRPW motif protein 1	Upregulation: Enhanced cell proliferation and survival, resulting in liver fibrosis
**P38 MAPK Signaling Pathway**<br><br>Increased pro-inflammatory cytokine release activates stress responses MAPKs, contributing to liver injury, inflammation, and fibrosis	MAPK14	Mitogen-activated protein kinase 14	Upregulation: Increased oxidative stress and inflammatory cytokines, causing cellular stress responses, promoting hepatocellular injury and liver fibrosis
	MAPK11	Mitogen-activated protein kinase 11	Upregulation: Increased oxidative stress and inflammatory cytokines, causing cellular stress responses, promoting hepatocellular injury and liver fibrosis
	MAPK12	Mitogen-activated protein kinase 12	Upregulation: Increased oxidative stress and inflammatory cytokines, causing cellular stress responses, promoting hepatocellular injury and liver fibrosis
	MKK3	Mitogen-Activated Protein Kinase Kinase 3	Upregulation: Increased oxidative stress and inflammatory cytokines, causing cellular stress responses, promoting hepatocellular injury and liver fibrosis
	MKK6	Mitogen-Activated Protein Kinase Kinase 6	Upregulation: Increased oxidative stress and inflammatory cytokines, causing cellular stress responses, promoting hepatocellular injury and liver fibrosis
**PI3K/AKT Signaling Pathway**<br><br>Signaling pathway involved in inflammation, oxidative stress, cell apoptosis, epithelial mesenchymal transformation, and autophagy	PI3K	Phosphoinositide 3-kinase	Upregulation: Increased oxidative stress and nutrient signaling enhances cell growth and survival, promoting liver fibrosis
	AKT1	AKT serine/threonine kinase 1	Upregulation: Increased oxidative stress and nutrient signaling enhances cell growth and survival, promoting liver fibrosis
	AKT2	AKT serine/threonine kinase 2	Upregulation: Increased oxidative stress and nutrient signaling enhances cell growth and survival, promoting liver fibrosis
	PTEN	Phosphatase and TENsin homolog	Upregulation: Increased oxidative stress and nutrient signaling enhances cell growth and survival, promoting liver fibrosis
	PDK1	Pyruvate dehydrogenase kinase 1	Upregulation: Increased oxidative stress and nutrient signaling enhances cell growth and survival, promoting liver fibrosis
	mTOR	Mechanistic Target of Rapamycin Kinase	Upregulation: Increased oxidative stress and nutrient signaling enhances cell growth and survival, promoting liver fibrosis
	RICTOR	Rapamycin-insensitive companion of mTOR	Upregulation: Increased oxidative stress and nutrient signaling enhances cell growth and survival, promoting liver fibrosis
	FOXO	Forkhead box O	Upregulation: Increased oxidative stress and nutrient signaling enhances cell growth and survival, promoting liver fibrosis
**Platelet Derived Growth Factor Signaling Pathway**<br><br>Disrupted cell growth and differentiation contributes to liver inflammation and fibrosis	ITGA2B	Integrin subunit alpha 2b	Upregulation: Increased integrins and platelet activation components enhance cell adhesion and signaling, increasing myofibroblast activity and ECM deposition, promoting liver fibrosis
	ITGB3	Integrin, beta 3	Upregulation: Increased integrins and platelet activation components enhance cell adhesion and signaling, increasing myofibroblast activity and ECM deposition, promoting liver fibrosis
	GP1BA	Glycoprotein Ib platelet subunit alpha	Upregulation: Increased integrins and platelet activation components enhance cell adhesion and signaling, increasing myofibroblast activity and ECM deposition, promoting liver fibrosis
	GP1BB	Glycoprotein Ib platelet subunit beta	Upregulation: Increased integrins and platelet activation components enhance cell adhesion and signaling, increasing myofibroblast activity and ECM deposition, promoting liver fibrosis
	GPIIB	Glycoprotein IIb	Upregulation: Increased integrins and platelet activation components enhance cell adhesion and signaling, increasing myofibroblast activity and ECM deposition, promoting liver fibrosis
	GPIIIA	Glycoprotein IIIa	Upregulation: Increased integrins and platelet activation components enhance cell adhesion and signaling, increasing myofibroblast activity and ECM deposition, promoting liver fibrosis
	PLCγ2	Phospholipase C, gamma 2	Upregulation: Increased integrins and platelet activation components enhance cell adhesion and signaling, increasing myofibroblast activity and ECM deposition, promoting liver fibrosis
**Rho GTPase Signaling Pathway**<br><br>Imparied actin cytoskeleton reorganization and cell proliferation promote liver inflammation and fibrosis	RHOA	Ras homolog family member A	Upregulation: Enhanced cell migration and myofibroblast activity, resulting in liver fibrosis
	RAC1	Ras-related C3 botulinum toxin substrate 1	Upregulation: Enhanced cell migration and myofibroblast activity, resulting in liver fibrosis
	CDC42	Cell division control protein 42 homolog	Upregulation: Enhanced cell migration and myofibroblast activity, resulting in liver fibrosis
	ROCK1	Rho-associated, coiled-coil containing protein kinase 1	Upregulation: Enhanced cell migration and myofibroblast activity, resulting in liver fibrosis
	ROCK2	Rho-associated coiled-coil containing protein kinase 2	Upregulation: Enhanced cell migration and myofibroblast activity, resulting in liver fibrosis
	PAK1	p21 (RAC1) activated kinase 1	Upregulation: Enhanced cell migration and myofibroblast activity, resulting in liver fibrosis
	PAK2	p21 (RAC1) activated kinase 2	Upregulation: Enhanced cell migration and myofibroblast activity, resulting in liver fibrosis
**Sonic Hedgehog (SHH) Pathway**<br><br>Impaired liver regeneration activates hepatic stellate cells (HSCs) and promotes inflammation and liver fibrosis	SHH	Sonic Hedgehog gene	Upregulation: Increased activation of myofibroblasts and ECM production, contributing to liver fibrosis
	PTCH1	Patched 1	Upregulation: Increased activation of myofibroblasts and ECM production, contributing to liver fibrosis
	SMO	Smoothened, Frizzled Class Receptor	Upregulation: Increased activation of myofibroblasts and ECM production, contributing to liver fibrosis
	GLI1	GLI Family Zinc Finger 1	Upregulation: Increased activation of myofibroblasts and ECM production, contributing to liver fibrosis
	GLI2	GLI Family Zinc Finger 2	Upregulation: Increased activation of myofibroblasts and ECM production, contributing to liver fibrosis
**Stress-Activated Protein Kinase (SAPK) Pathway**<br><br>Increased pro-inflammatory cytokine release activates stress responses MAPKs, contributing to liver injury, inflammation, and fibrosis	JNK1	Mitogen-Activated Protein Kinase 8 (MAPK8)	Upregulation: Increased oxidative stress and inflammatory cytokines, resulting in cellular stress responses and leading to hepatocellular injury and liver fibrosis
	JNK2	Mitogen-Activated Protein Kinase 9 (MAPK9)	Upregulation: Increased oxidative stress and inflammatory cytokines, resulting in cellular stress responses and leading to hepatocellular injury and liver fibrosis
	P38	Mitogen-Activated Protein Kinase 14 (MAPK14, also called p38α)	Upregulation: Increased oxidative stress and inflammatory cytokines, resulting in cellular stress responses and leading to hepatocellular injury and liver fibrosis
	ATF2	Activating Transcription Factor 2	Upregulation: Increased oxidative stress and inflammatory cytokines, resulting in cellular stress responses and leading to hepatocellular injury and liver fibrosis

	c-Jun	Jun Proto-Oncogene, AP-1 Transcription Factor Subunit	Upregulation: Increased oxidative stress and inflammatory cytokines, resulting in cellular stress responses and leading to hepatocellular injury and liver fibrosis
**T-Cell Receptor Signaling Pathway**<br><br>HSC activation and macrophage accumulation promotes liver inflammatoin and fibrosis	CD3	CD3 Complex (subunits: CD3D, CD3E, CD3G, CD247)	Upregulation: Increased T cell signaling results in inflammation and immune-mediated hepatocellular damage, promoting liver fibrosis
	ZAP70	Zeta Chain of T-Cell Receptor Associated Protein Kinase 70	Upregulation: Increased T cell signaling results in inflammation and immune-mediated hepatocellular damage, promoting liver fibrosis
	LAT	Linker for Activation of T Cells	Upregulation: Increased T cell signaling results in inflammation and immune-mediated hepatocellular damage, promoting liver fibrosis
	LCK	LCK Proto-Oncogene, Src Family Tyrosine Kinase	Upregulation: Increased T cell signaling results in inflammation and immune-mediated hepatocellular damage, promoting liver fibrosis
	ITK	IL2-Inducible T Cell Kinase	Upregulation: Increased T cell signaling results in inflammation and immune-mediated hepatocellular damage, promoting liver fibrosis
**TGF- β Signaling Pathway**<br><br>Activation of fibroblasts and stimulating them to differentiate into myofibroblasts, which produce ECM proteins	TGF-β1	Transforming Growth Factor Beta 1	Upregulation: Increased TGF-β and SMAD proteins promote myofibroblast activation and ECM production, leading to liver fibrosis
	TGF-β2	Transforming Growth Factor Beta 2	Upregulation: Increased TGF-β and SMAD proteins promote myofibroblast activation and ECM production, leading to liver fibrosis
	TGF-β3	Transforming Growth Factor Beta 3	Upregulation: Increased TGF-β and SMAD proteins promote myofibroblast activation and ECM production, leading to liver fibrosis
	SMAD2	SMAD Family Member 2	Upregulation: Increased TGF-β and SMAD proteins promote myofibroblast activation and ECM production, leading to liver fibrosis
	SMAD3	SMAD Family Member 3	Upregulation: Increased TGF-β and SMAD proteins promote myofibroblast activation and ECM production, leading to liver fibrosis
	SMAD4	SMAD Family Member 4	Upregulation: Increased TGF-β and SMAD proteins promote myofibroblast activation and ECM production, leading to liver fibrosis
	SMAD7	SMAD Family Member 7	Upregulation: Increased TGF-β and SMAD proteins promote myofibroblast activation and ECM production, leading to liver fibrosis
	TGFBR1	Transforming Growth Factor Beta Receptor 1	Upregulation: Increased TGF-β and SMAD proteins promote myofibroblast activation and ECM production, leading to liver fibrosis
	TGFBR2	Transforming Growth Factor Beta Receptor 2	Upregulation: Increased TGF-β and SMAD proteins promote myofibroblast activation and ECM production, leading to liver fibrosis
**Toll-like Receptor (TLR) Signaling Pathway**<br><br>Accumulation of PAMPs and DAMPs activates TLR signalling, inducing production of inflammatory and fibrogenic cytokines, leading to liver inflammation and fibrosis	TLR2	Toll-Like Receptor 2	Upregulation: Increased TLRs and adaptor proteins result in inflammation and immune-mediated hepatocellular damage, contributing to liver fibrosis
	TLR3	Toll-Like Receptor 3	Upregulation: Increased TLRs and adaptor proteins result in inflammation and immune-mediated hepatocellular damage, contributing to liver fibrosis
	TLR4	Toll-Like Receptor 4	Upregulation: Increased TLRs and adaptor proteins result in inflammation and immune-mediated hepatocellular damage, contributing to liver fibrosis
	TLR9	Toll-Like Receptor 9	Upregulation: Increased TLRs and adaptor proteins result in inflammation and immune-mediated hepatocellular damage, contributing to liver fibrosis
	MYD88	Myeloid Differentiation Primary Response 88	Upregulation: Increased TLRs and adaptor proteins result in inflammation and immune-mediated hepatocellular damage, contributing to liver fibrosis
	IRAK1	Interleukin-1 Receptor-Associated Kinase 1	Upregulation: Increased TLRs and adaptor proteins result in inflammation and immune-mediated hepatocellular damage, contributing to liver fibrosis
	TRAF6	TNF Receptor-Associated Factor 6	Upregulation: Increased TLRs and adaptor proteins result in inflammation and immune-mediated hepatocellular damage, contributing to liver fibrosis
	TRIF	TIR-Domain-Containing Adapter-Inducing Interferon-β	Upregulation: Increased TLRs and adaptor proteins result in inflammation and immune-mediated hepatocellular damage, contributing to liver fibrosis
**TRAIL Signaling Pathway**<br><br>HSC activation increases apoptosis via elevated death cell receptor expression, promoting liver inflammation and fibrosis	TRAIL	TNF-Related Apoptosis-Inducing Ligand	Upregulation: Increased TRAIL and death receptors, activating extrinsic apoptotic pathway, leading to apoptosis of hepatocytes and immune cells, promoting liver fibrosis
	DR4	Death Receptor 4	Upregulation: Increased TRAIL and death receptors, activating extrinsic apoptotic pathway, leading to apoptosis of hepatocytes and immune cells, promoting liver fibrosis
	DR5	Death Receptor 5	Upregulation: Increased TRAIL and death receptors, activating extrinsic apoptotic pathway, leading to apoptosis of hepatocytes and immune cells, promoting liver fibrosis
	FADD	Fas-Associated Death Domain Protein	Upregulation: Increased TRAIL and death receptors, activating extrinsic apoptotic pathway, leading to apoptosis of hepatocytes and immune cells, promoting liver fibrosis
	CASP8	Caspase 8	Upregulation: Increased TRAIL and death receptors, activating extrinsic apoptotic pathway, leading to apoptosis of hepatocytes and immune cells, promoting liver fibrosis
**Unfolded Protein Response (UPR) Pathway**<br><br>Accumulation of misfolded or unfolded proteins leads to ER stress, promoting apoptosis and contributing to inflammation and liver fibrosis	ATF4	Activating Transcription Factor 4	Upregulation: Increased endoplasmic reticulum stress, resulting in cellulr stress and apoptosis, promoting liver fibrosis
	EIF2AK3	Eukaryotic Translation Initiation Factor 2 Alpha Kinase 3	Upregulation: Increased endoplasmic reticulum stress, resulting in cellulr stress and apoptosis, promoting liver fibrosis
	IRE1	Inositol-Requiring Enzyme 1	Upregulation: Increased endoplasmic reticulum stress, resulting in cellulr stress and apoptosis, promoting liver fibrosis
	XBP1	X-Box Binding Protein 1	Upregulation: Increased endoplasmic reticulum stress, resulting in cellulr stress and apoptosis, promoting liver fibrosis
**Wnt-B-Catenin Signaling Pathway**<br><br>Promotes fibrosis by inducing expression of fibrogenic genes	WNT1	Wnt Family Member 1	Upregulation: Increased myofibroblast activation and ECM production, contributing to liver fibrosis
	WNT3A	Wnt Family Member 3A	Upregulation: Increased myofibroblast activation and ECM production, contributing to liver fibrosis
	β-catenin	Catenin Beta 1	Upregulation: Increased myofibroblast activation and ECM production, contributing to liver fibrosis
	LRP5	Low Density Lipoprotein Receptor-Related Protein 5	Upregulation: Increased myofibroblast activation and ECM production, contributing to liver fibrosis
	LRP6	Low Density Lipoprotein Receptor-Related Protein 6	Upregulation: Increased myofibroblast activation and ECM production, contributing to liver fibrosis
	GSK3β	Glycogen Synthase Kinase 3 Beta	Upregulation: Increased myofibroblast activation and ECM production, contributing to liver fibrosis
	AXIN	Axis Inhibitor	Upregulation: Increased myofibroblast activation and ECM production, contributing to liver fibrosis
	APC	Adenomatous Polyposis Coli	Upregulation: Increased myofibroblast activation and ECM production, contributing to liver fibrosis
