# Supplementary material for: E-cigarette vaping is associated with pro-fibrotic gene expression in kidney and liver tissues
Source: J Mol Med (Berl). 2026 Jul 31;104(1):99. doi: 10.1007/s00109-026-02699-1 (PMC13424587; doi:10.1007/s00109-026-02699-1)
Supplement: Supplementary file 3 — Supplementary Material 3 [file 109_2026_2699_MOESM3_ESM.pdf]

Pathway Name and Function	Gene Abbreviation	Gene Name	E-cigarette Exposure Effect on Gene Function
**ECM and Cytoskeletal Pathway**<br><br>Production of proteins that contribute to ECM and cytoskeleton structure and function; crucial in maintaining tissue integrity and cellular function	FN1	Fibronectin 1	Upregulation: Induces extracellular matrix remodeling, promoting fibrosis
	VIM	Vimentin	Upregulation: Induces epithelial-mesenchymal transition, promoting fibrosis
	COL1A1	Collagen, type I, alpha 1 chain	Upregulation: Increased reactive oxygen species (ROS) production and inflammatory cytokines, resulting in excessive extracellular matrix (ECM) deposition, promoting fibrosis
	COL1A2	Collagen type I alpha 2 chain	Upregulation: Increased reactive oxygen species (ROS) production and inflammatory cytokines, resulting in excessive extracellular matrix (ECM) deposition, promoting fibrosis
	COL4A1	Collagen type IV alpha 1 chain	Upregulation: Increased reactive oxygen species (ROS) production and inflammatory cytokines, resulting in excessive extracellular matrix (ECM) deposition, promoting fibrosis
	ITGA5	Integrin subunit alpha 5	Upregulation: Increased integrin and focal adhesion components enhance cell adhesion and migration, increasing myofibroblast activity and ECM deposition, promoting fibrosis
	ITGB1	Integrin beta-1	Upregulation: Increased integrin and focal adhesion components enhance cell adhesion and migration, increasing myofibroblast activity and ECM deposition, promoting fibrosis
**JAK/STAT Signaling Pathway**<br><br>Signaling pathway that mediates various biological processes such as cell proliferation, differentiation, apoptosis, and immune responses, due to which, prolonged activation can promote fibrosis and inflammation.	JAK1	Janus kinase 1	Upregulation: Increased production of cytokines and growth factors, resulting in inflammation and fibrosis
	JAK2	Janus kinase 2	Upregulation: Increased production of cytokines and growth factors, resulting in inflammation and fibrosis
	JAK3	Janus kinase 3	Upregulation: Increased production of cytokines and growth factors, resulting in inflammation and fibrosis
	SOCS1	Suppressor of cytokine signaling 1	Upregulation: Increased production of cytokines and growth factors, resulting in inflammation and fibrosis
	SOCS3	Suppressor of cytokine signaling 3	Upregulation: Increased production of cytokines and growth factors, resulting in inflammation and fibrosis
	STAT1	Signal Transducer and Activator of Transcription 1	Upregulation: Increased production of cytokines and growth factors, resulting in inflammation and fibrosis
	STAT3	Signal Transducer and Activator of Transcription 3	Upregulation: Increased production of cytokines and growth factors, resulting in inflammation and fibrosis
	TYK2	Tyrosine kinase 2	Upregulation: Increased production of cytokines and growth factors, resulting in inflammation and fibrosis
**RAS/MAPK Signaling Pathway**<br><br>Pathway involved in regulation of cellular processes involved in renal fibrosis, such as activation and proliferation of myofibroblasts or extracellular matrix protein accumulation	BRAF	B-Raf proto-oncogene, serine/threonine kinase	Upregulation: Increased ROS and inflammatory cytokines enhance cell proliferation and cell survival, promoting fibrosis
	MAPK1	Mitogen-Activated Protein Kinase 1	Upregulation: Promotes fibrosis by influencing MAPK signaling downstream
	MAPK3	Mitogen-Activated Protein Kinase 3	Upregulation: Promotes fibrosis by influencing MAPK signaling downstream
	RAF1	RAF-1 proto-oncogene, serine/threonine kinase	Upregulation: Increased ROS and inflammatory cytokines enhance cell proliferation and cell survival, promoting fibrosis
**NF-κB Signaling Pathway**<br><br>Signaling pathway known to promote fibrosis by causing hyperactivation of NFKB, triggering recruitment and activation of immune cells, resulting in inflammation and oxidative stress.	IKBKA	CHUK - component of inhibitor of nuclear factor kappa B kinase complex	Upregulation: Increased inflammatory cytokines and oxidative stress lead to inflammation, promoting fibrosis
	IKBKB	Inhibitor of nuclear factor kappa B kinase subunit beta	Upregulation: Increased inflammatory cytokines and oxidative stress lead to inflammation, promoting fibrosis
	IKBKG	Inhibitor of nuclear factor kappa-B kinase regulatory subunit gamma	Upregulation: Increased inflammatory cytokines and oxidative stress lead to inflammation, promoting fibrosis
	IL1B	Interleukin 1 Beta	Upregulation: Promotes fibrosis via NF-κB signaling activation
	IL6	Interleukin 6	Upregulation: Promotes fibrosis via NF-κB signaling activation, resulting in inflammation and fibrogenic responses
	NFKB1	Nuclear factor kappa B subunit 1	Upregulation: Increased inflammatory cytokines and oxidative stress lead to inflammation, promoting fibrosis
	NFKB2	nuclear factor kappa B subunit 2	Upregulation: Increased inflammatory cytokines and oxidative stress lead to inflammation, promoting fibrosis
	RELA	RELA proto-oncogene, NF-kB subunit	Upregulation: Increased inflammatory cytokines and oxidative stress lead to inflammation, promoting fibrosis
	RELB	RELB proto-oncogene, NF-kB subunit	Upregulation: Increased inflammatory cytokines and oxidative stress lead to inflammation, promoting fibrosis
	TNF	Tumor Necrosis Factor	Upregulation: induces fibrosis via NF-κB signaling activation, causing inflammation and ECM remodeling
	TRAF6	TNF Receptor-Associated Factor 6	Upregulation: Involved in fibrosis through activating NF-κB and mediating NF-κB signaling
**Notch Signaling Pathway**<br><br>Involved in development of fibrosis by facilitating myofibroblast formation and epithelial-mesenchymal transition	DLL1	Delta like canonical Notch ligand 1	Upregulation: Enhanced cell proliferation and survival, resulting in fibrosis
	DLL4	Delta like canonical Notch ligand 4	Upregulation: Enhanced cell proliferation and survival, resulting in fibrosis
	JAG1	Jagged 1	Upregulation: Promotes fibrosis by facilitating Notch signaling as ligand for Notch receptors
	NOTCH1	Notch receptor 1	Upregulation: Enhanced cell proliferation and survival, resulting in fibrosis
	NOTCH2	Notch receptor 2	Upregulation: Enhanced cell proliferation and survival, resulting in fibrosis
	NOTCH3	Notch receptor 3	Upregulation: Enhanced cell proliferation and survival, resulting in fibrosis
	NOTCH4	Notch receptor 4	Upregulation: Enhanced cell proliferation and survival, resulting in fibrosis

**PI3K/AKT Signaling Pathway**<br><br>Signaling pathway involved in inflammation, oxidative stress, cell apoptosis, epithelial mesenchymal transformation, and autophagy	AKT1	AKT serine/threonine kinase 1	Upregulation: Promotes fibrosis by facilitating TGF- β dependent ECM production and myofibroblast differentiation
	AKT2	AKT serine/threonine kinase 2	Upregulation: Promotes fibrosis by facilitating TGF- β dependent ECM production and myofibroblast differentiation
	MTOR	Mechanistic target of rapamycin kinase	Upregulation: Increased oxidative stress and nutrient signaling promote cell growth and survival, contributing to fibrosis
	PTEN	Phosphatase and TENsin homolog	Downregulation: Promotes fibrosis due to decreased negative regulation of PI3K/AKT signaling
**TGF-β Signaling Pathway**<br><br>Activation of fibroblasts and stimulating them to differentiate into myofibroblasts, which produce ECM proteins	SMAD2	SMAD Family Member 2	Upregulation: Increased TGF-β and SMAD proteins promote myofibroblast activation and ECM production, leading to fibrosis
	SMAD3	SMAD Family Member 3	Upregulation: Increased TGF-β and SMAD proteins promote myofibroblast activation and ECM production, leading to fibrosis
	SMAD4	SMAD Family Member 4	Upregulation: Increased TGF-β and SMAD proteins promote myofibroblast activation and ECM production, leading to fibrosis
	SMAD7	SMAD Family Member 7	Upregulation: Increased TGF-β and SMAD proteins promote myofibroblast activation and ECM production, leading to fibrosis
	TGF-β1	Transforming Growth Factor Beta 1	Upregulation: Increased TGF-β and SMAD proteins promote myofibroblast activation and ECM production, leading to fibrosis
	TGF-β2	Transforming Growth Factor Beta 2	Upregulation: Increased TGF-β and SMAD proteins promote myofibroblast activation and ECM production, leading to fibrosis
	TGF-β3	Transforming Growth Factor Beta 3	Upregulation: Increased TGF-β and SMAD proteins promote myofibroblast activation and ECM production, leading to fibrosis
	TGFBR1	Transforming Growth Factor Beta Receptor 1	Upregulation: Increased TGF-β and SMAD proteins promote myofibroblast activation and ECM production, leading to fibrosis
	TGFBR2	Transforming Growth Factor Beta Receptor 2	Upregulation: Increased TGF-β and SMAD proteins promote myofibroblast activation and ECM production, leading to fibrosis
**Wnt/β-Catenin Signaling Pathway**<br><br>Promotes fibrosis by inducing expression of fibrogenic genes	APC	Adenomatous Polyposis Coli	Upregulation: Increased myofibroblast activation and ECM production, contributing to fibrosis
	AXIN1	Axis Inhibitor 1	Upregulation: Increased myofibroblast activation and ECM production, contributing to fibrosis
	LRP5	Low Density Lipoprotein Receptor-Related Protein 5	Upregulation: Increased myofibroblast activation and ECM production, contributing to fibrosis
	LRP6	Low Density Lipoprotein Receptor-Related Protein 6	Upregulation: Increased myofibroblast activation and ECM production, contributing to fibrosis
	WNT1	Wnt Family Member 1	Upregulation: Increased myofibroblast activation and ECM production, contributing to fibrosis
