# Supplementary material for: E-cigarette vaping is associated with pro-fibrotic gene expression in kidney and liver tissues
Source: J Mol Med (Berl). 2026 Jul 31;104(1):99. doi: 10.1007/s00109-026-02699-1 (PMC13424587; doi:10.1007/s00109-026-02699-1)
Supplement: Supplementary file 4 — Supplementary Material 4 [file 109_2026_2699_MOESM4_ESM.pdf]

ID	Term Description	Fold Enrichment	P-value	Up-regulated	Down-regulated	Cluster	Status
hsa04146	Peroxisome	6.8139	6.28E-14	Hao2	Abcd3, Scp2, Amacr, Crat, Pecr, Ephx2, Prdx1	1	Representative
hsa00120	Primary bile acid biosynthesis	8.5173	5.59E-03		Amacr, Scp2	1	Member
hsa03083	Polycomb repressive complex	6.6246	1.27E-10	Asxl2, Ogt, Phc3, Crebbp, Ep300, Mga	Skp1	2	Representative
hsa04330	Notch signaling pathway	4.6196	1.54E-08	Tle3, Crebbp, Ep300, Spen		2	Member
hsa04068	FoxO signaling pathway	3.3788	5.26E-08	Foxo3, Pik3R1, Crebbp, Ep300, Sgk1	Bcl6	2	Member
hsa04066	HIF-1 signaling pathway	4.1717	5.72E-07	Crebbp, Ep300, Pik3R1, Flt1	Ldha, Pgk1	2	Member
hsa05211	Renal cell carcinoma	5.4950	1.19E-05	Crebbp, Ep300, Epas1, Pik3R1	Met	2	Member
hsa05215	Prostate cancer	5.2414	1.56E-05	Crebbp, Ep300, Creb3L2, Pik3R1, Tmprss2	Hsp90Ab1, Gstp1	2	Member
hsa04350	TGF-beta signaling pathway	2.7255	3.85E-05	Crebbp, Ep300	Id2, Skp1	2	Member
hsa05206	MicroRNAs in cancer	1.4943	3.98E-05	Crebbp, Ep300, Pik3R1, Mdm4	Met	2	Member
hsa04310	Wnt signaling pathway	2.1427	7.61E-05	Tle3, Crebbp, Ep300, Chd8	Skp1	2	Member
hsa04919	Thyroid hormone signaling pathway	1.7931	9.74E-05	Pik3R1, Crebbp, Ep300		2	Member
hsa04935	Growth hormone synthesis, secretion and action	3.1840	1.09E-04	Adcy6, Creb3L2, Pik3R1, Crebbp, Ep300		2	Member
hsa04520	Adherens junction	4.2061	1.16E-04	Crebbp, Ep300, Ptprb, Lmo7	Met	2	Member
hsa04922	Glucagon signaling pathway	3.4413	2.00E-04	Creb3L2, Crebbp, Ep300	Acaca, Ldha	2	Member
hsa05203	Viral carcinogenesis	1.8292	2.85E-04	Creb3L2, Pik3R1, Crebbp, Ep300		2	Member
hsa05161	Hepatitis B	1.8292	2.85E-04	Creb3L2, Pik3R1, Crebbp, Ep300		2	Member
hsa05152	Tuberculosis	2.0401	3.01E-04	Crebbp, Ep300	Lamp2, Ctsd, Hspd1	2	Member
hsa04630	JAK-STAT signaling pathway	1.7035	5.44E-04	Lifr, Crebbp, Ep300, Pik3R1		2	Member
hsa05167	Kaposi sarcoma-associated herpesvirus infection	1.1549	8.14E-04	Pik3R1, Crebbp, Ep300		2	Member
hsa04024	cAMP signaling pathway	2.6462	9.07E-04	Adcy6, Pik3R1, Rapgef3, Creb3L2, Pde4C, Crebbp, Ep300	Ppp1Ca	2	Member
hsa04720	Long-term potentiation	4.4681	1.01E-03	Crebbp, Ep300, Rapgef3	Ppp1Ca	2	Member
hsa04110	Cell cycle	2.3496	2.40E-03	Crebbp, Ep300, Atrx, Ppp2R5A	Skp1	2	Member
hsa04916	Melanogenesis	2.8995	3.72E-03	Crebbp, Ep300, Creb3L2, Adcy6		2	Member
hsa05164	Influenza A	1.7814	7.56E-03	Crebbp, Ep300, Pik3R1, Tmprss2		2	Member
hsa05166	Human T-cell leukemia virus 1 infection	1.7035	1.83E-02	Adcy6, Creb3L2, Pik3R1, Crebbp, Ep300		2	Member
hsa03250	Viral life cycle - HIV-1	2.6207	4.78E-02	Crebbp, Ep300		2	Member
hsa04510	Focal adhesion	4.7624	8.09E-09	Itga1, Flna, Mylk, Pik3R1, Col4A1, Col4A2, Col4A3, Col4A4, Lama5, Flt1, Dock1	Ppp1Ca, Met	3	Representative
hsa04512	ECM-receptor interaction	6.4894	1.29E-02	Lama5, Col4A1, Col4A2, Col4A3, Col4A4, Itga1, Agrn	Cd36	3	Member
hsa03082	ATP-dependent chromatin remodeling	4.0082	9.33E-07	Arid1A, Bptf, Ino80D, Srcap, Ep400		4	Representative
hsa04142	Lysosome	4.2922	3.29E-06	Dmxl2	Ctsb, Ctsd, Hexb, Lipa, Ppt2, Lamp2, M6Pr	5	Representative
hsa04210	Apoptosis	2.2341	1.08E-02	Pik3R1, Map3K5	Ctsb, Ctsd	5	Member
hsa04140	Autophagy - animal	2.2713	1.51E-02	Pik3R1, Vmp1	Ctsd, Ctsb, Lamp2	5	Member
hsa04152	AMPK signaling pathway	4.8670	3.77E-06	Pik3R1, Foxo3, Ppp2R5A, Creb3L2	Adipor2, Acaca, Fasn, Cd36	6	Representative
hsa04936	Alcoholic liver disease	4.0082	3.96E-04	Foxo3, Map3K5	Adipor2, Acaca, Fasn, Cyp2E1, Aldh3A2, Acadvl	6	Member
hsa00620	Pyruvate metabolism	7.7430	2.69E-03		Me1, Ldha, Aldh3A2, Acaca, Grhpr	6	Member
hsa04910	Insulin signaling pathway	2.1128	3.86E-03	Pik3R1	Fasn, Ppp1Ca, Acaca	6	Member
hsa00061	Fatty acid biosynthesis	8.5173	4.35E-03		Fasn, Acaca	6	Member
hsa00640	Propanoate metabolism	7.0488	1.89E-02		Acaca, Ldha, Suclg2	6	Member
hsa00310	Lysine degradation	8.6722	1.85E-05	Kmt2A, Ash1L, Kmt2E, Kmt2C, Kmt2D	Hadh, Aldh3A2	7	Representative
hsa01212	Fatty acid metabolism	9.5394	2.31E-04		Fasn, Hadhb, Hadh, Scp2, Acaca, Acadvl, Ppt2	7	Member
hsa00071	Fatty acid degradation	6.4894	4.59E-02		Hadh, Hadhb, Acadvl, Aldh3A2	7	Member
hsa00190	Oxidative phosphorylation	6.2512	3.48E-05	Nd6	Cox7C, Cox7A2, Cyc1, Uqcrc2, Uqcrfs1, Atp6V1E1, [illegible]	8	Representative
hsa04260	Cardiac muscle contraction	4.4828	3.81E-05		Cox7A2, Cox7C, Cyc1, Uqcrc2, Uqcrfs1	8	Member
hsa05020	Prion disease	3.5551	6.42E-05	Nd6, Pik3R1, Creb3L2	Hspa8, Sdhb, Sdhc, Sdhd, Cyc1, Uqcrc2, Uqcrfs1, Cox[illegible]	8	Member
hsa04932	Non-alcoholic fatty liver disease	6.7179	1.01E-04	Mlxipl, Pik3R1, Map3K5	Adipor2, Cyp2E1, Cox7A2, Cox7C, Sdhb, Sdhc, Sdhd[illegible]	8	Member
hsa05016	Huntington disease	3.4333	1.13E-04	Nd6, Crebbp, Ep300, Creb3L2, Map3K5	Sdhb, Sdhc, Sdhd, Cyc1, Uqcrc2, Uqcrfs1, Cox7A2, [illegible]	8	Member
hsa05208	Chemical carcinogenesis - reactive oxygen species	5.2957	3.44E-04	Pik3R1, Nd6, Foxo3, Map3K5	Met, Sdhb, Sdhc, Sdhd, Cyc1, Uqcrc2, Uqcrfs1, Cox7[illegible]	8	Member
hsa05415	Diabetic cardiomyopathy	5.4201	1.11E-03	Nd6, Pik3R1	Cd36, Sdhb, Sdhc, Sdhd, Cyc1, Uqcrc2, Uqcrfs1, Cox[illegible]	8	Member
hsa04714	Thermogenesis	4.5194	1.70E-03	Creb3L2, Adcy6, Arid1A, Map3K5, Nd6	Cox7A2, Cox7C, Cyc1, Sdhb, Sdhc, Sdhd, Uqcrc2, U[illegible]	8	Member
hsa01200	Carbon metabolism	6.3681	1.95E-03	Hao2	Pgk1, Taldo1, Sdhb, Sdhc, Sdhd, Suclg2, Glyctk, Esd, [illegible]	8	Member
hsa05012	Parkinson disease	3.3019	3.03E-03	Nd6, Map3K5	Sdhb, Sdhc, Sdhd, Cyc1, Uqcrc2, Uqcrfs1, Cox7A2, [illegible]	8	Member
hsa05100	Bacterial invasion of epithelial cells	3.0510	4.13E-05	Dock1, Pik3R1	Met	9	Representative
hsa04960	Aldosterone-regulated sodium reabsorption	4.6992	5.25E-05	Sgk1, Pik3R1		9	Member
hsa04915	Estrogen signaling pathway	4.3960	5.84E-05	Creb3L2, Adcy6, Pik3R1, Fkbp5	Hsp90Ab1, Fkbp4, Hspa8, Ctsd	9	Member
hsa04668	TNF signaling pathway	2.0239	5.98E-05	Creb3L2, Pik3R1, Map3K5		9	Member
hsa04213	Longevity regulating pathway - multiple species	5.0473	1.17E-04	Foxo3, Pik3R1, Adcy6	Hspa8	9	Member
hsa04015	Rap1 signaling pathway	2.4335	2.21E-04	Flt1, Rapgef3, Rap1Gap, Adcy6, Pik3R1, Sipa1L3	Met	9	Member
hsa04962	Vasopressin-regulated water reabsorption	6.3385	2.52E-04	Adcy6, Creb3L2, Aqp2, Dync2H1		9	Member
hsa04014	Ras signaling pathway	1.5846	3.20E-04	Pik3R1, Flt1, Ralgapa1	Met, Rgl1	9	Member
hsa05417	Lipid and atherosclerosis	2.4842	3.37E-04	Pik3R1, Map3K5	Cd36, Xbp1, Hspa8, Hsp90Ab1, Hspd1	9	Member

hsa04810	Regulation of actin cytoskeleton	1.8927	5.39E-04	Mylk, Arhgef7, Pik3R1, Dock1, Itga1	Ppp1Ca	9	Member
hsa05223	Non-small cell lung cancer	3.0972	6.53E-04	Foxo3, Pik3R1	Met	9	Member
hsa01524	Platinum drug resistance	3.8936	7.81E-04	Pik3R1, Map3K5	Slc31A1, Gstp1	9	Member
hsa01521	EGFR tyrosine kinase inhibitor resistance	2.9202	7.81E-04	Pik3R1, Foxo3	Met	9	Member
hsa04722	Neurotrophin signaling pathway	1.8583	2.05E-03	Pik3R1, Foxo3, Map3K5		9	Member
hsa05205	Proteoglycans in cancer	1.4345	2.69E-03	Pik3R1, Flna	Ppp1Ca, Met	9	Member
hsa04725	Cholinergic synapse	2.3769	2.84E-03	Adcy6, Creb3L2, Pik3R1		9	Member
hsa04931	Insulin resistance	4.1296	3.17E-03	Pik3R1, Mlxipl, Ogt, Creb3L2	Cd36, Ppp1Ca	9	Member
hsa04750	Inflammatory mediator regulation of TRP channels	2.1980	3.60E-03	Adcy6, Pik3R1	Ppp1Ca	9	Member
hsa05418	Fluid shear stress and atherosclerosis	2.0648	4.23E-03	Pik3R1, Map3K5	Gstp1, Hsp90Ab1	9	Member
hsa05225	Hepatocellular carcinoma	2.1700	5.94E-03	Pik3R1, Arid1A	Met, Gstp1, Txnrd1	9	Member
hsa04150	mTOR signaling pathway	1.9750	6.03E-03	Pik3R1, Sgk1, Fnip2	Atp6V1E1	9	Member
hsa05135	Yersinia infection	2.1804	6.38E-03	Dock1, Arhgef7, Arhgef28, Pik3R1		9	Member
hsa04218	Cellular senescence	1.3906	6.46E-03	Pik3R1, Foxo3	Ppp1Ca	9	Member
hsa05017	Spinocerebellar ataxia	2.1631	6.53E-03	Atxn2L, Map3K5, Pik3R1	Xbp1	9	Member
hsa04611	Platelet activation	2.3295	7.17E-03	Pik3R1, Mylk, Adcy6	Ppp1Ca	9	Member
hsa04926	Relaxin signaling pathway	4.0767	7.17E-03	Pik3R1, Creb3L2, Col4A1, Col4A2, Col4A3, Col4A4, Adcy6		9	Member
hsa04211	Longevity regulating pathway	4.1548	8.25E-03	Foxo3, Pik3R1, Adcy6, Creb3L2	Adipor2	9	Member
hsa05213	Endometrial cancer	2.5237	9.46E-03	Foxo3, Pik3R1		9	Member
hsa04666	Fc gamma R-mediated phagocytosis	1.5664	9.85E-03	Pik3R1, Dock1		9	Member
hsa04360	Axon guidance	0.8016	1.12E-02	Pik3R1	Met	9	Member
hsa05230	Central carbon metabolism in cancer	3.1940	1.58E-02	Pik3R1	Ldha, Met	9	Member
hsa04917	Prolactin signaling pathway	2.0648	1.73E-02	Pik3R1, Foxo3		9	Member
hsa05131	Shigellosis	1.4196	2.30E-02	Dock1, Pik3R1, Foxo3	Skp1	9	Member
hsa04062	Chemokine signaling pathway	1.1748	2.34E-02	Pik3R1, Adcy6, Foxo3		9	Member
hsa05171	Coronavirus disease - COVID-19	2.1680	2.42E-02	Tmprss2, Pik3R1	Rps3, Rps4X, Rps14, Rpl13, Rpl41	9	Member
hsa04930	Type II diabetes mellitus	1.7931	3.26E-02	Pik3R1		9	Member
hsa05170	Human immunodeficiency virus 1 infection	0.7699	3.27E-02	Pik3R1	Skp1	9	Member
hsa05163	Human cytomegalovirus infection	1.0272	3.49E-02	Pik3R1, Adcy6, Creb3L2		9	Member
hsa04929	GnRH secretion	1.5846	4.18E-02	Pik3R1		9	Member
hsa04973	Carbohydrate digestion and absorption	1.5846	4.18E-02	Pik3R1		9	Member
hsa04914	Progesterone-mediated oocyte maturation	2.2713	4.36E-02	Adcy6, Pik3R1	Hsp90Ab1	9	Member
hsa04934	Cushing syndrome	1.9468	4.91E-02	Creb3L2, Adcy6, Kmt2A, Kmt2D		9	Member
hsa04141	Protein processing in endoplasmic reticulum	4.6670	6.50E-05	Map3K5, Herpud1	Rpn1, Xbp1, Hspa8, Dnaja1, Skp1, Hsp90Ab1, Tram1,	10	Representative
hsa05132	Salmonella infection	1.8416	3.53E-02	Flna, Dync2H1	Hsp90Ab1, Rps3, Skp1, M6Pr	10	Member
hsa04010	MAPK signaling pathway	1.2905	2.72E-04	Flt1, Map3K5, Flna	Met, Hspa8	11	Representative
hsa05202	Transcriptional misregulation in cancer	3.9821	3.25E-04	Fus, Flt1, Pax8, Tmprss2, Kmt2A, Jmjd1C	Id2, Met, Bcl6	11	Member
hsa04020	Calcium signaling pathway	0.8849	2.80E-02	Flt1, Mylk	Met	11	Member
hsa03040	Spliceosome	4.2922	4.69E-04	Snrnp70, Rbm25, Sf3B1, Srsf2, Srsf5, Acin1, Fus	Hspa8	12	Representative
hsa04612	Antigen processing and presentation	3.4647	2.00E-03		Ctsb, Hsp90Ab1, Hspa8	12	Member
hsa05134	Legionellosis	4.0883	4.41E-02		Hspd1, Eef1G, Hspa8	12	Member
hsa03020	RNA polymerase	2.7255	3.01E-02	Polr3E		13	Representative
