# Supplementary material for: E-cigarette vaping is associated with pro-fibrotic gene expression in kidney and liver tissues
Source: J Mol Med (Berl). 2026 Jul 31;104(1):99. doi: 10.1007/s00109-026-02699-1 (PMC13424587; doi:10.1007/s00109-026-02699-1)
Supplement: Supplementary file 5 — Supplementary Material 5 [file 109_2026_2699_MOESM5_ESM.pdf]

gene_name	gene_symbol	Source	Kidney_Air_1	Kidney_Air_2	Kidney_Air_3	Kidney_Air_4	Kidney_Air_5	Kidney_Air_6	Kidney_EV_1	Kidney_EV_2	Kidney_EV_3	Kidney_EV_4	Kidney_EV_5	Kidney_EV_6
ENSMUSG00000000740	Rpl13	kidney_ev_air	82.75	60.92	91.58	98.61	68.33	100.86	98.6	104.87	121.63	90.96	137.21	135.79
ENSMUSG00000002944	Cd36	kidney_ev_air	233	151.52	231.58	206.07	203.36	230.88	269.04	273.82	314.86	210.5	366.83	289.91
ENSMUSG00000020154	Ptprb	kidney_ev_air	191.26	103.96	135.83	161.06	128.81	162.59	99.95	115.38	137.24	95.86	133.63	134.36
ENSMUSG00000020538	Srebf1	liver_veh_air	76.75	55.35	50.77	80.95	45.86	46.76	52.36	73.64	63.35	47.42	119.37	62.11
ENSMUSG00000020593	Lpin1	liver_veh_air	109.03	75.71	152.68	118.7	94.06	169.39	126.76	94.14	117.37	77.93	132.11	145.92
ENSMUSG00000023150	Ivns1abp	liver_veh_air	534.67	352.5	498.12	541.68	497.92	504.82	504.94	487.46	535.45	354.12	441.25	461.77
ENSMUSG00000027559	Car3	kidney_ev_air; kidney_veh_air	14.04	21.62	29.53	16.83	28.83	13.6	50.45	100.9	96.71	38.65	318.41	125.31
ENSMUSG00000027947	Il6ra	liver_veh_air	10.06	6.73	10.64	8.77	7.53	8.52	4.47	7.15	8.01	5.12	6.97	7.11
ENSMUSG00000028410	Dnaja1	kidney_ev_air	122.34	86.02	118.12	113.22	115.89	115.83	142.28	152.7	170.1	115.56	159.3	146.24
ENSMUSG00000031765	Mt1	liver_veh_air	49.74	29.41	38.68	52.44	57.43	88	49.15	37.58	39.16	35.29	35.1	55.3
ENSMUSG00000036885	Arhgef26	liver_veh_air	9.25	5.37	10.92	5.64	6.5	8.08	5.51	4.2	6.36	5.35	6.86	7.37
ENSMUSG00000045672	Col27a1	kidney_veh_air	112.97	88.65	84.83	125.72	113.03	98.34	99.73	88.77	111.29	88.18	80.96	98.05
ENSMUSG00000048756	Foxo3	kidney_veh_air	213.57	133	212.64	184.44	146.43	272.22	201.93	137.69	154.67	112.2	145.12	192.28
ENSMUSG00000050445	Cyp8b1	liver_ev_air	0.09	0	0.36	0.12	0.09	0	0.13	0	0.14	0.11	2.77	0.16
ENSMUSG00000071796	6820431F20Rik	kidney_ev_air	197.3	112.12	144.39	144.5	150.78	172.1	144.97	130.88	138.71	93.7	111.85	118.6
ENSMUSG00000093930	Hmgcs1	liver_ev_air	475.04	275.9	478.51	450.66	331.45	407.02	450.28	410.49	508.17	394.26	433.57	538.63
ENSMUSG00000102307		kidney_ev_air	200.6	108.43	153.95	166.81	153.87	149.54	145.02	120.07	126.41	93.47	94.86	110.54
ENSMUSG00000105703		liver_veh_air	3644.33	2692.62	5921.19	3234.67	2741.26	3824.76	3826.3	2317.54	2902.52	3061.26	2793.33	4083.6
ENSMUSG00000110779		kidney_ev_air	346.44	193.51	260.56	283.8	239.55	306.56	274.94	222.03	218.12	155.69	171.65	199.97
ENSMUSG00000111481		kidney_ev_air	277.56	147.37	203.95	229.58	203.28	235.41	206.88	168.08	166.62	141.23	137.87	155.05
ENSMUSG00000113673		kidney_ev_air	142.38	73.6	104.71	111	94.82	106.77	100.42	79.84	77.05	57.72	66.62	73.52

Kidney_VEH_1	Kidney_VEH_2	Kidney_VEH_3	Kidney_VEH_4	Kidney_VEH_5	Liver_Air_1	Liver_Air_2	Liver_Air_3	Liver_Air_4	Liver_Air_5	Liver_Air_6	Liver_EV_1	Liver_EV_2	Liver_EV_3	Liver_EV_4	Liver_EV_5	Liver_EV_6	Liver_VEH_1	Liver_VEH_2
102.29	110.07	97.02	107.45	114.73	40.05	35.56	2.04	24.08	29.28	11.89	10.56	33.86	24.46	2.95	13.32	9.3	16.04	14.57
238.79	249.13	268.58	259.64	298.55	37.22	28.94	9.17	33.09	17.41	23.84	27.73	16.9	19.08	3.57	13.87	37.34	22.24	19.41
137.64	158.14	143.82	144.65	151.59	244.47	248.9	80.42	198.2	196.43	171.87	166.63	157.31	186.73	54.6	138.68	188.39	156.9	198.2
99.17	128.88	113.5	101.52	65.19	340.81	273.03	30.66	182.65	223.95	175.89	194.3	354.51	248.73	81.75	373.69	310.28	342.45	519.72
102.01	114.18	142.49	98.04	111.15	839.18	831.65	397.95	141.16	192.6	1027.59	227.82	49.38	271.4	71.84	76.35	666.4	43.18	56.29
447.4	489.64	494.39	541.36	520.58	121.23	132.38	58.64	94.01	128.98	133.5	94.59	62.39	64.54	35.61	67.63	115.58	42.95	56.6
72.39	68.09	449.43	56.33	68.9	2266.06	2102.55	374.53	1736.64	1458.33	1437.28	679.94	1289.06	1517.34	271.44	1140.37	997.67	1395.79	1920.98
7.85	10.07	9.85	7.65	9.38	115.14	122.98	35.74	59.39	70.43	85.47	61.25	49.75	42.18	19.75	23.27	67.56	16.61	24.12
117.15	125.42	133.03	140.51	139.09	75.69	56.16	17.29	56.08	48.82	60.49	39.9	70.82	59.15	10.41	38.04	48.14	37.1	43.46
34.02	30.32	35.75	35.13	46.24	60.24	39.54	39.77	100.92	87.46	85.88	177.19	31.52	11.27	9.28	6.32	65.07	3.15	2.48
4.54	7.26	6.38	5.09	5.4	124.3	153.05	53.44	116.07	90.02	183.87	132.7	63.35	106.59	29.4	36.73	104.34	56.58	42.1
56.82	77.81	98.35	72.48	89.92	61.36	86	29.9	55.47	85.97	100.01	64.23	53.59	79.34	33.98	127.84	122.14	77.41	99.69
120.08	141.8	129.56	135.31	162.98	167.61	161.85	35.57	97.49	110.67	123.85	112.72	130.49	122.14	28.46	94.74	106.4	62.88	93.49
0	0	0.09	0.08	0.12	209.99	494.19	81.24	224.18	251.1	123.1	56.78	123.08	124.86	21.75	45.45	86.11	99.7	155.12
136.83	155.51	151.95	152.76	143.44	135.7	125.92	26.22	97.57	104.12	93	80.87	112.31	88.26	22	74.08	66.75	71.78	88.84
485.11	446.37	429.65	455.1	506.5	452.64	624.1	42.46	215.9	152.46	97.71	81.47	90.17	112.1	17.36	45.52	113.84	77.35	212.09
123.58	185.38	142.28	145.72	157.42	93.79	95.25	19.74	90.01	69.68	61	63.34	92.63	74.57	19.81	45.24	39.09	57.82	58.4
4155.31	3265.98	3972.21	2627.88	3662.73	409.38	604.32	492.56	384.73	274.63	1372.48	440.14	188.19	188.9	68.58	122.48	475.39	113.66	163.73
219.01	316.46	279.07	266.3	263.76	297.5	283.56	58.11	255.25	229.38	189.85	179.21	312.01	223.15	52.47	165.6	127.82	195.35	190.77
169.95	260	182.57	186.09	217.74	191.43	199.44	37.84	170.12	155.02	117.76	136.27	190.27	150.75	30.03	95.5	86.67	123.06	122.69
93.4	120.56	100.36	95.9	100.94	88.34	98.4	33.52	110.49	99.6	89.44	84.03	102.92	87.33	29.46	74.22	79.87	76.79	89.96

Liver_VEH_3	Liver_VEH_4	Liver_VEH_5	Mean_Kidney_Air	Mean_Kidney_EV	Mean_Kidney_VEH	Mean_Liver_Air	Mean_Liver_EV	Mean_Liver_VEH	SD_Kidney_Air	SD_Kidney_EV	SD_Kidney_VEH	SD_Liver_Air	SD_Liver_EV	SD_Liver_VEH
10.27	69.45	23.32	83.84	114.84	106.31	23.82	15.74	26.73	16.34	19.59	6.87	14.48	11.33	24.34
21.1	27.94	22.86	209.4	287.49	262.94	24.94	19.75	22.71	31.33	51.97	22.83	10.39	11.64	3.2
178.27	183.19	179.37	147.25	119.4	147.17	190.05	148.72	179.19	30.68	18.4	7.88	61.45	49.74	14.79
468.71	372.25	312	59.41	69.71	101.65	204.5	260.54	403.03	15.49	25.99	23.54	105.04	110.09	87.8
93.32	58.58	167.83	119.93	115.7	113.57	571.69	227.2	83.84	35.39	25.29	17.45	375.83	233.82	50.48
69.97	55.8	51.35	488.29	464.16	498.67	111.46	73.39	55.33	69.14	63.13	35.46	29.73	27.89	9.81
1492.28	2522.62	1639.18	20.74	121.74	143.03	1562.57	982.64	1794.17	7.14	101.72	171.39	671.53	447.74	452.93
30.86	38.35	33.88	8.71	6.47	8.96	81.52	43.96	28.76	1.48	1.36	1.13	33.39	19.53	8.54
41.58	93.18	43.5	111.9	147.7	131.04	52.42	44.41	51.76	13.04	18.58	9.78	19.4	20.72	23.3
9.6	8.2	21.92	52.62	41.93	36.29	68.97	50.11	9.07	20.08	8.35	5.95	26.24	66.05	7.82
44.11	41.44	73.84	7.63	5.94	5.73	120.12	78.85	51.61	2.19	1.15	1.08	45.9	41.9	13.87
95.5	23.39	62.71	103.92	94.5	79.08	69.79	80.19	71.74	15.93	10.75	16.04	25.71	37.76	30.81
108.35	122.73	114.97	193.72	157.32	137.95	116.17	99.16	100.48	50.84	34.03	16.11	48.31	36.78	23.61
111.16	245.05	127.72	0.11	0.55	0.06	230.63	76.34	147.75	0.13	1.09	0.05	144.38	42.31	58.25
74.13	86.26	75.8	153.53	123.12	148.1	97.09	74.04	79.36	28.8	18.94	7.75	38.5	29.92	7.66
102.07	300.77	159.83	403.1	455.9	464.55	264.21	76.74	170.42	83.01	56.51	30.9	226.6	38.29	89.68
68.01	53.73	59.01	155.53	115.06	150.88	71.58	55.78	59.39	29.73	19.73	22.8	28.97	26.27	5.24
134.73	123.59	136.06	3676.47	3164.09	3536.82	589.68	247.28	134.35	1191.6	665.9	609.81	399.05	169.49	18.78
210.26	205.34	187.64	271.74	207.07	268.92	218.94	176.71	197.87	53.33	42.18	34.96	87.74	87.66	9.63
145.78	109.27	121.87	216.19	162.62	203.27	145.27	114.92	124.53	43.28	25.03	36.27	60.13	56.21	13.2
100.67	49.67	70.33	105.55	75.86	102.23	86.63	76.3	77.48	22.47	14.42	10.71	27.23	24.9	19.48
