# Supplementary material for: E-cigarette vaping is associated with pro-fibrotic gene expression in kidney and liver tissues
Source: J Mol Med (Berl). 2026 Jul 31;104(1):99. doi: 10.1007/s00109-026-02699-1 (PMC13424587; doi:10.1007/s00109-026-02699-1)
Supplement: Supplementary file 6 — Supplementary Material 6 [file 109_2026_2699_MOESM6_ESM.pdf]

gene_name	gene_symbol	Source	Kidney_Air_1	Kidney_Air_2	Kidney_Air_3	Kidney_Air_4	Kidney_Air_5	Kidney_Air_6	Kidney_EV_1	Kidney_EV_2	Kidney_EV_3	Kidney_EV_4	Kidney_EV_5	Kidney_EV_6	Kidney_VEH_1
ENSMUSG00000031502	Col4a1	Canonical_Fibrosis_Marker	299.95	181.51	247.2	304.8	248.33	233.54	203.88	248.12	285.33	180.85	277.31	221.03	260.51
ENSMUSG00000026193	Fn1	Canonical_Fibrosis_Marker	69.43	50.61	55.53	74.63	83.21	57.35	38.69	60.3	79.52	46.22	111.89	56.15	62.69

Kidney_VEH_2	Kidney_VEH_3	Kidney_VEH_4	Kidney_VEH_5	Liver_Air_1	Liver_Air_2	Liver_Air_3	Liver_Air_4	Liver_Air_5	Liver_Air_6	Liver_EV_1	Liver_EV_2	Liver_EV_3	Liver_EV_4	Liver_EV_5	Liver_EV_6	Liver_VEH_1	Liver_VEH_2	Liver_VEH_3
295.34	241.99	276.13	262.1	51.41	39.09	9.75	41.17	26.19	23.21	23.97	35.09	40.13	5.64	28.22	24.92	30.74	37.51	26.82
75.15	53.35	89.51	64.57	2278.99	1484.39	345.68	1867.54	1962.45	1389.77	2085.04	2463.41	1622.57	489.6	1437.99	1397.13	1546.44	1997.92	1770.71

Liver_VEH_4	Liver_VEH_5	Mean_Kidney_Air	Mean_Kidney_EV	Mean_Kidney_VEH	Mean_Liver_Air	Mean_Liver_EV	Mean_Liver_VEH	SD_Kidney_Air	SD_Kidney_EV	SD_Kidney_VEH	SD_Liver_Air	SD_Liver_EV	SD_Liver_VEH
38.35	31.28	252.55	236.09	267.21	31.8	26.33	32.94	45.68	41.43	19.86	14.95	11.89	4.88
2347.47	1585.45	65.13	65.46	69.05	1554.8	1582.62	1849.6	12.64	26.66	13.81	675.66	675.25	330.82
