# Supplementary material for: E-cigarette vaping is associated with pro-fibrotic gene expression in kidney and liver tissues
Source: J Mol Med (Berl). 2026 Jul 31;104(1):99. doi: 10.1007/s00109-026-02699-1 (PMC13424587; doi:10.1007/s00109-026-02699-1)
Supplement: Supplementary file 7 — Supplementary Material 7 [file 109_2026_2699_MOESM7_ESM.docx]

**Title:** E-cigarette Vaping is Associated with Pro-Fibrotic Gene Expression in Kidney and Liver Tissues

**Supplementary Table Legends**

**Supplemental Table 1:** Kidney Gene List for A priori Gene Set Analysis. A curated list of 109 genes with established roles in renal fibrosis, assembled from published literature prior to transcriptomic data collection. Includes gene symbols and Ensembl gene IDs (GRCm39). This gene set was used to conduct within-set false discovery rate (FDR) correction in the kidney a priori differential expression analysis.

**Supplemental Table 2:** Liver Gene List for A priori Gene Set Analysis. A curated list of genes with established roles in hepatic fibrosis, assembled from published literature prior to transcriptomic data collection. Includes gene symbols and Ensembl gene IDs (GRCm39). This gene set was used to conduct within-set FDR correction in the liver a priori differential expression analysis.

**Supplemental Table 3:** Common Genes from Final Kidney and Liver Gene Lists Used for A priori Gene Set Analysis. Genes present in both the kidney and liver a priori fibrosis gene sets, representing shared molecular mechanisms of organ fibrosis. Includes gene symbols and Ensembl gene IDs (GRCm39).

**Supplementary Table 4:** All Biological Pathways Enriched in the Kidney and Liver. Complete results from gene set enrichment analysis performed using the pathfindR R package with the KEGG pathway database. Includes all significantly enriched pathways (adjusted p < 0.05) for each of the four primary comparisons: kidney E-cig vs Air, kidney Vehicle vs Air, liver E-cig vs Air, and liver Vehicle vs Air, with columns for KEGG pathway ID, pathway description, fold enrichment, occurrence, support, lowest adjusted p-value, constituent upregulated genes, constituent downregulated genes, pathway cluster assignment, and cluster representative status. Functionally similar pathways are grouped by gene-overlap clustering; representative pathways are flagged.

**Supplementary Table 5:** Normalized CPM Expression Values for Each Individual Mouse for All Significantly Differentially Expressed Genes (Table 1). Values shown are counts per million (CPM) normalized to library size for each individual animal (kidney: n = 6 Air, 6 E-cig, 5 Vehicle; liver: n = 6 Air, 6 E-cig, 5 Vehicle), with group means and standard deviations.

**Supplementary Table 6:** Normalized CPM Expression Values for Each Individual Mouse for Canonical Pro-Fibrotic Marker Genes Detected Above the Expression Threshold. Of 26 canonical fibrosis markers queried, 2 (Col4a1, Fn1) were detected at CPM > 100 in ≥ 2 samples and are shown. The remaining 24 markers (Col1a1, Col1a2, Col3a1, Acta2, Tgfb1, Tgfb2,

Tgfb3, Vim, Mmp2, Mmp9, Timp1, Pdgfrb, Snai1, Snai2, Ccn2, Postn, Lox, Fsp1, S100a4, Tnc, Tgfbr1, Tgfbr2, Aldh1a7, Ctgf) were not detected above the expression filter, consistent with the absence of overt fibrotic remodeling in these tissues at the time of harvest.
